# Histone H3K18 and Ezrin Lactylation Promote Renal Dysfunction in Sepsis‐Associated Acute Kidney Injury

**DOI:** 10.1002/advs.202307216

**Published:** 2024-05-20

**Authors:** Jiao Qiao, Yuan Tan, Hongchao Liu, Boxin Yang, Qian Zhang, Qi Liu, Wenyuan Sun, Zhongxin Li, Qingchen Wang, Weimin Feng, Shuo Yang, Liyan Cui

**Affiliations:** ^1^ Institute of Medical Technology Peking University Health Science Center Beijing 100191 China; ^2^ Department of Laboratory Medicine Peking University Third Hospital Beijing 100191 China; ^3^ Core Unit of National Clinical Research Center for Laboratory Medicine Peking University Third Hospital Beijing 100191 China

**Keywords:** ezrin, glycolysis, lactylation, RhoA, sepsis‐associated acute kidney injury

## Abstract

Histone lactylation is a metabolic stress‐related histone modification. However, the role of histone lactylation in the development of sepsis‐associated acute kidney injury (SA‐AKI) remains unclear. Here, histone H3K18 lactylation (H3K18la) is elevated in SA‐AKI, which is reported in this study. Furthermore, this lactate‐dependent histone modification is enriched at the promoter of Ras homolog gene family member A (RhoA) and positively correlated with the transcription. Correction of abnormal lactate levels resulted in a reversal of abnormal histone lactylation at the promoter of RhoA. Examination of related mechanism revealed that histone lactylation promoted the RhoA/Rho‐associated protein kinase (ROCK) /Ezrin signaling, the activation of nuclear factor‐κB (NF‐κB), inflammation, cell apoptosis, and aggravated renal dysfunction. In addition, Ezrin can undergo lactylation modification. Multiple lactylation sites are identified in Ezrin and confirmed that lactylation mainly occurred at the K263 site. The role of histone lactylation is revealed in SA‐AKI and reportes a novel post‐translational modification in Ezrin. Its potential role in regulating inflammatory metabolic adaptation of renal proximal tubule epithelial cells is also elucidated. The results provide novel insights into the epigenetic regulation of the onset of SA‐AKI.

## Graphical abstract

H3K18la is elevated and positively correlated with the RhoA transcription in SA‐AKI. Mechanistically, histone lactylation promotes the RhoA/ROCK/Ezrin signaling pathway, NF‐κB activation, inflammation, and cell apoptosis and aggravates renal dysfunction. In addition, exogenous lactate promotes Ezrin lactylation, which mainly occurs at the K263 site.

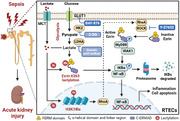

## 1 Introduction

Sepsis‐associated acute kidney injury (SA‐AKI) is commonly characterized as sepsis or septic shock affecting the kidneys, leading to a gradual deterioration in kidney function, as per the criteria outlined by the Global Renal Disease Prognosis Organization (KDIGO) for acute kidney injury (AKI). This definition also involves the exclusion of alternative causes of kidney injury.^[^ ^1^, ^2^ ^]^ Extrapolating from incidence rates in the United States, Adhikari et al. determined that as many as 190 000 cases of sepsis are reported worldwide each year.^[^ ^3^ ^]^ Compared with non‐septic AKI, SA‐AKI is strongly associated with adverse clinical outcomes.^[^ ^4^, ^5^, ^6^ ^]^

Renal tubular epithelial cells (RTECs) are the most metabolically active cells in the kidney and are sensitive to sepsis‐related damage. In both the cecal ligation and puncture (CLP) model and human SA‐AKI, RTECs may undergo an initial shift to glycolysis, converting pyruvate to lactic acid. This represents an inefficient mechanism for producing ATP. Importantly, serum lactate has been recognized as a biomarker of sepsis prognosis, and elevated serum lactate levels are positively associated with sepsis‐related mortality.^[^ ^7^ ^]^ Lactate, a derivative of aerobic glycolysis, is known to directly enhance the release of inflammatory factors, such as tumor necrosis factor α (TNF‐α), interleukin‐6 (IL‐6), and interleukin‐1β (IL‐1β),^[^ ^8^ ^]^ and a large number of inflammatory factors that lead to worsening of renal function, apoptotic cell death, and sublethal damage (loss of cell polarity, reduced expression of tight junction protein, and bioenergy interference).^[^ ^9^ ^]^ In contrast, inhibiting aerobic glycolysis and inducing oxidative phosphorylation (OXPHOS) can reduce susceptibility to AKI and significantly improve survival, improving inflammation‐related conditions such as sepsis and multiple sclerosis.^[^ ^10^, ^11^ ^]^ Therefore, reprogramming glycolysis in the proximal tubule epithelium may be a promising strategy for maintaining or restoring kidney function. This modulation may regulate the potential role of the inflammatory metabolic adaptation of the proximal tubule epithelium, thereby offering a potential avenue for improving inflammation and apoptosis.

Long‐established protein post‐translational modifications (PTMs) include acetylation, ubiquitination, and methylation of lysine residues and phosphorylation and glycosylation of serine or threonine residues.^[^ ^12^ ^]^ These modifications form a part of the hierarchy of epigenetic regulatory mechanisms. Glycolysis‐derived lactate has been identified as a substrate for histone lactylation and activates downstream gene transcription directly.^[^ ^13^, ^14^ ^]^ Interestingly, histone lactylation plays an important role as an epigenetic regulator mechanism in pathogenesis. For example, histone lactylation regulates the M1/2 polarization of macrophages and promotes the transformation of macrophages from an inflammatory state to a repaired state.^[^ ^15^ ^]^ Histone lactylation promotes the expression of m6A reader protein YTHDF2 to promote the development of tumors.^[^ ^16^ ^]^ Glis1 promotes somatic reprogramming through an epigenome‐metabolome‐epigenome signaling cascade.^[^ ^17^ ^]^ However, the role of histone lactylation in the development of SA‐AKI remains unclear. Because of the metabolic reprogramming of RTECs during the early stage of SA‐AKI, histone lactylation in RTECs is likely to be abnormal, and it is necessary to explore the potential function of histone lactylation in the development of SA‐AKI. Moreover, PTMs can widely regulate the function of proteins, shape cell phenotypes, and play a vital role in the endogenous regulation of Ezrin. Examination of PTMs of Ezrin would help enrich the understanding of the Ezrin regulation mechanism.

Therefore, in this study, we report for the first time the potential role of H3K18la and lactylation on the FREM domain of Ezrin in SA‐AKI, identifying Ezrin as a lactate substrate. We provide evidence that SA‐AKI promotes an increase in lactate and H3K18la and activates RhoA/ROCK1/Ezrin signaling and Ezrin K263 lactylation, leading to downstream inflammation and apoptosis. Inhibition of GLUT1 and Ezrin K263 site mutations (K263R) alleviated inflammation and apoptosis, providing a new mechanism and potential target for delaying renal dysfunction.

## 2 Results

### 2.1 Histone Lactylation Increases Rapidly in Renal Tissue and Tubular Epithelial Cells After SA‐AKI

**Figure 1:**
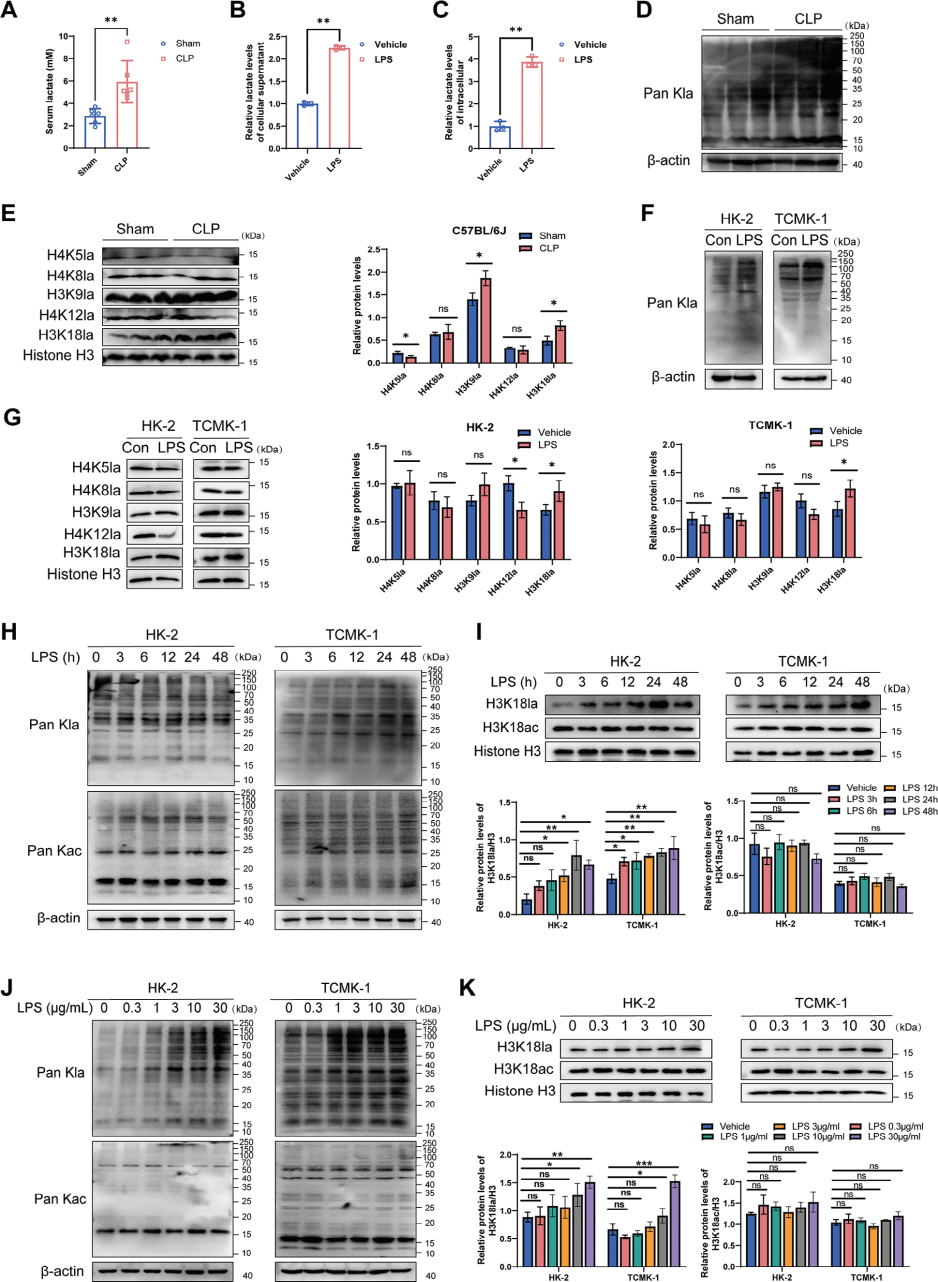
Increased histone lactylation in SA‐AKI mice and RTECs induced by LPS. A) Lactate levels in the serum of CLP model and Sham group mice (n  = 6 mice per group). B) The relative levels of lactate in the supernatant of HK‐2 cells treated with LPS, n  =  3 independent experiments. C) Changes in intracellular lactate levels in HK‐2 cells after LPS treatment, n  =  3 independent experiments. D) Western blotting analysis of Pan Kla in the total proteins extracted from the kidney tissues of CLP and Sham group mice (n  = 6 mice per group); β‐actin served as an internal control; normalized corresponding densitometric analyses are shown as a histogram. E) Western blotting analysis of site‐specific histone lactylation in the total proteins extracted from the kidney tissue of CLP and Sham group mice (n  = 6 mice per group); Histone H3 served as an internal control; normalized corresponding densitometric analyses are shown as a histogram. F) Western blotting analysis of Pan Kla in HK‐2 cells and TCMK‐1 cells; Histone H3 served as an internal control. G) Western blotting analysis of site‐specific histone lactylation in HK‐2 cells and TCMK‐1 cells. Histone H3 served as an internal control. H) The Pan Kla and Pan Kac western blotting analysis of HK‐2 cells and TCMI‐1 cells was performed at 0, 3, 6, 12, 24, and 48 h after LPS treatment. I) Time gradient‐based western blotting analysis of H3K18la and H3K18ac in HK‐2 cells and TCMK‐1 cells after LPS treatment. J) The Pan Kla and Pan Kac western blotting analysis of HK‐2 cells and TCMI‐1 cells treated with LPS at 0, 0.3, 1, 3, 10, and 30 µg mL^−1^. K) Concentration gradient‐based western blotting analysis of H3K18la and H3K18ac in HK‐2 cells and TCMK‐1 cells after LPS treatment. *P < 0.05, **P < 0.01, and ***P < 0.001; ns, not significant.

Although the pathophysiological mechanisms of SA‐AKI, especially the inflammatory response, microvascular dysfunction, and metabolic recombination, have been studied, the role of histone lactylation as epigenetic modification in the development of SA‐AKI remains unclear. Given the metabolic reprogramming from OXPHOS to glycolysis in RTECs during SA‐AKI,^[^ ^18^, ^19^ ^]^ we found that GLUT1 protein expression levels were significantly increased after stimulation of HK‐2 cells with LPS (Figure S1A,B, Supporting Information). Therefore, we first examined lactate levels in the peripheral blood of CLP mice. Colorimetric measurements showed that serum lactate levels were significantly higher in mice in the CLP group compared to those in matched Sham mice (**Figure** **1**A). The results were consistent with previously reported clinical observations indicating that patients with SA‐AKI have higher lactate concentrations than healthy controls.^[^ ^20^, ^21^, ^22^ ^]^ Similarly, lactate concentration in the supernatant and cell lysate of HK‐2 cells was higher in the LPS‐stimulated group than in the vehicle group (Figure 1B,C). Since lactate acts as a precursor that can stimulate histone lactylation,^[^ ^23^ ^]^ we hypothesized that histone lactylation might change in the context of SA‐AKI. Western blot analysis showed increased levels of Pan Kla, H3K9la, and H3K18la in total kidney proteins extracted from mice in the CLP group compared to that in the Sham group (Figure 1D,E). Consistent with observations in mice, levels of Pan Kla and H3K18la were significantly increased in HK‐2 cells and TCMK‐1 cells (Figure 1F,G). To further evaluate the lactylation‐related changes in RTECs in vitro after LPS stimulation at different time and concentration gradients, western blot analysis was performed. The results showed that the lactylation level increased as early as 3 h after LPS stimulation in the early stage, with HK‐2 cells reaching a peak at 12 h and TCMK‐1 cells reaching a peak at 6 h. Lactylation decreased to baseline levels within 48 h of LPS stimulation. In contrast, the level of acetylation did not change significantly (Figure 1H). Subsequently, we examined the lactylation of H3K18la. The results showed that H3K18la levels gradually increased in HK‐2 cells and began to decline at 24 h. It remained elevated compared to baseline and the difference was statistically significant. In comparison, TCMK‐1 cells maintained a high level of H3K18la until 48 h (Figure 1I). In response to stimulation with different concentrations of LPS, the levels of global lactylation and H3K18la levels showed a similar trend, and with the increase of LPS concentration, the global lactylation and H3K18la levels were gradually upregulated. However, the levels of global acetylation and H3K18ac were not significantly changed (Figure 1J,K). These results all support increased lactate and histone lactylation in the context of SA‐AKI.

### 2.2 Inhibition of Histone Lactylation Alleviates LPS‐Induced RTEC Damage

**Figure 2:**
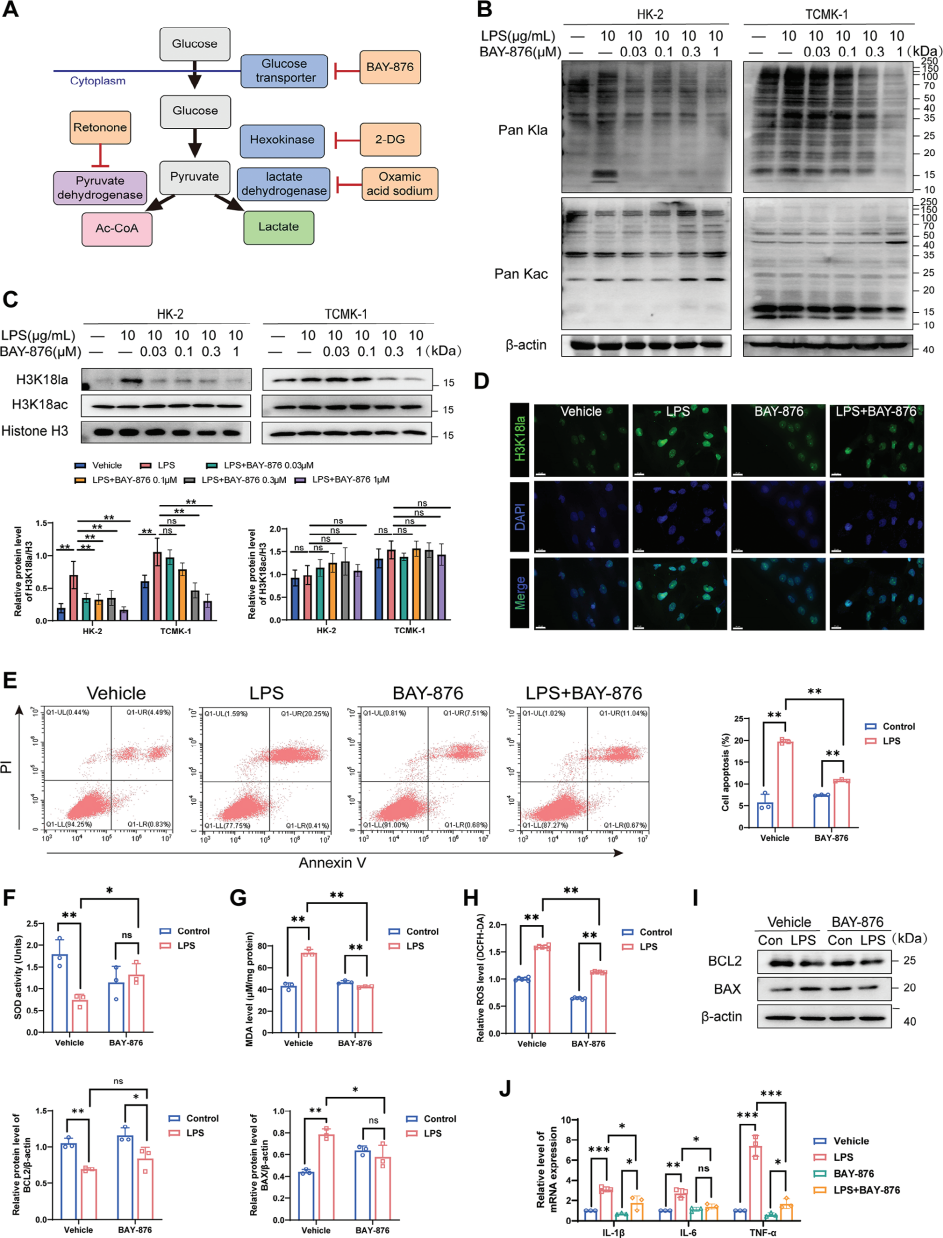
Inhibition of GLUT1 can decrease the level of histone lactylation and inhibit LPS‐induced RTECs injury. A) Schematic diagram of histone lactylation inhibition. B) Pan Kla and Pan Kac levels were detected in HK‐2 cells and TCMK‐1 cells cultured in different concentrations of BAY‐876 and 10 µg/mL of LPS for 24 h by western blotting analysis. C) H3K18la and H3K18ac levels were detected in HK‐2 cells and TCMK‐1 cells cultured in different concentrations of BAY‐876 and 10 µg/mL of LPS for 24 h by western blotting analysis. D) Immunofluorescence staining for H3K18la (green) and 4′,6‐diamidino‐2‐phenylindole (DAPI; blue) in HK‐2 cells (scale bar = 20 µm). E) Flow cytometry and corresponding quantitative analysis of the extent of HK‐2 cell apoptosis by Annexin V/PI staining, n  =  3 independent experiments. F,G,H) Detection of SOD activities (F), MDA levels (G), and ROS levels (H) in HK‐2 cells after different treatments, n  =  3 independent experiments. (I) Western blotting analysis of BCL2 and BAX protein levels in HK‐2 cells incubated with 10 µg/mL LPS ± 1 µM BAY‐876 for 24 h, n  =  3 independent experiments. (J) Detection of IL (interleukin)−1, IL (interleukin)−6, and TNF‐α (tumor necrosis factor‐α) mRNA expression levels by real‐time qPCR, n  =  3 independent experiments. ^*^ *p* <0.05, ^**^ *p* <0.01, and ^***^ *p* <0.001; ns, not significant.

Lactate production depends on a balance between glycolysis and mitochondrial metabolism. We first tested whether inhibition of glycolysis‐related enzyme activity reduced histone lactylation levels in RTECs (**Figure** **2**A). In this paper, we applied two inhibitors: 1) Glucose transporter GLUT1 inhibitor, BAY‐876; 2) Glycolysis inhibitors, the non‐metabolizable glucose analogs 2‐deoxyd‐glucose (2‐DG) and Oxamic acid sodium. Notably, both classes of inhibitors achieved significant reductions in lactic acid within RTECs (Figure S2A,B, Supporting Information) and reductions in global lactylation and H3K18la levels (Figure 2B,C; Figure S2C,D, Supporting Information). Immunofluorescence also confirmed changes in H3K18la levels in HK‐2 cells (Figure 2D). And we introduced siRNAs specific to GLUT1 and LDHA/LDHB.^[^ ^16^ ^]^ to further validate the role of histone lactylation in SA‐AKI. We observed that silencing GLUT1 or LDHA/LDHB with LPS treatment significantly impaired histone lactylation, increased cell apoptosis, and exacerbated damage. Reintroducing lactate into GLUT1 or LDHA/LDHB‐deficient cells with LPS treatment successfully elevated histone lactylation levels and partially restored the extent of cell damage (Figure S2E,F, Supporting Information). Conversely, mitochondrial electron transport chain Complex I inhibitors (Rotenone) mediated cellular glycolysis and increased intracellular lactate levels, as well as lactate and H3K18la levels (Figure S2G,H, Supporting Information).

Next, we evaluated whether Histone lactylation inhibition mitigated LPS‐induced RTEC damage in vitro. Flow cytometry (FCM) showed that BAY‐876 reduced LPS‐induced apoptosis (P<0.01, Figure 2E). Upon measuring the expression of oxidative stress markers in HK‐2 cell supernatant, we observed that LPS up‐regulated MDA and down‐regulated SOD compared to the corresponding levels observed in the control group. In contrast, the BAY+LPS group inhibited LPS‐induced oxidative stress factors (*p *<0.05, Figure 2F,G). LPS then induced ROS expression, while BAY‐876 prevented LPS‐mediated oxidative stress (*p *<0.01, Figure 2H). Similarly, Western blot showed that compared with the control group, the level of apoptosis marker BCL2‐associated X (BAX) was increased, whereas the addition of BAY‐876 inhibited LPS‐induced kidney injury and apoptosis (Figure 2I). The expression of inflammatory factors in HK‐2 cells was also confirmed by Real‐time qPCR (Figure 2J). Thus, BAY‐876 alleviated LPS‐induced inflammation, oxidative stress, and apoptosis of renal tubular epithelial cells.

### 2.3 Identification of Potential Downstream Targets of H3K18la Regulation

**Figure 3:**
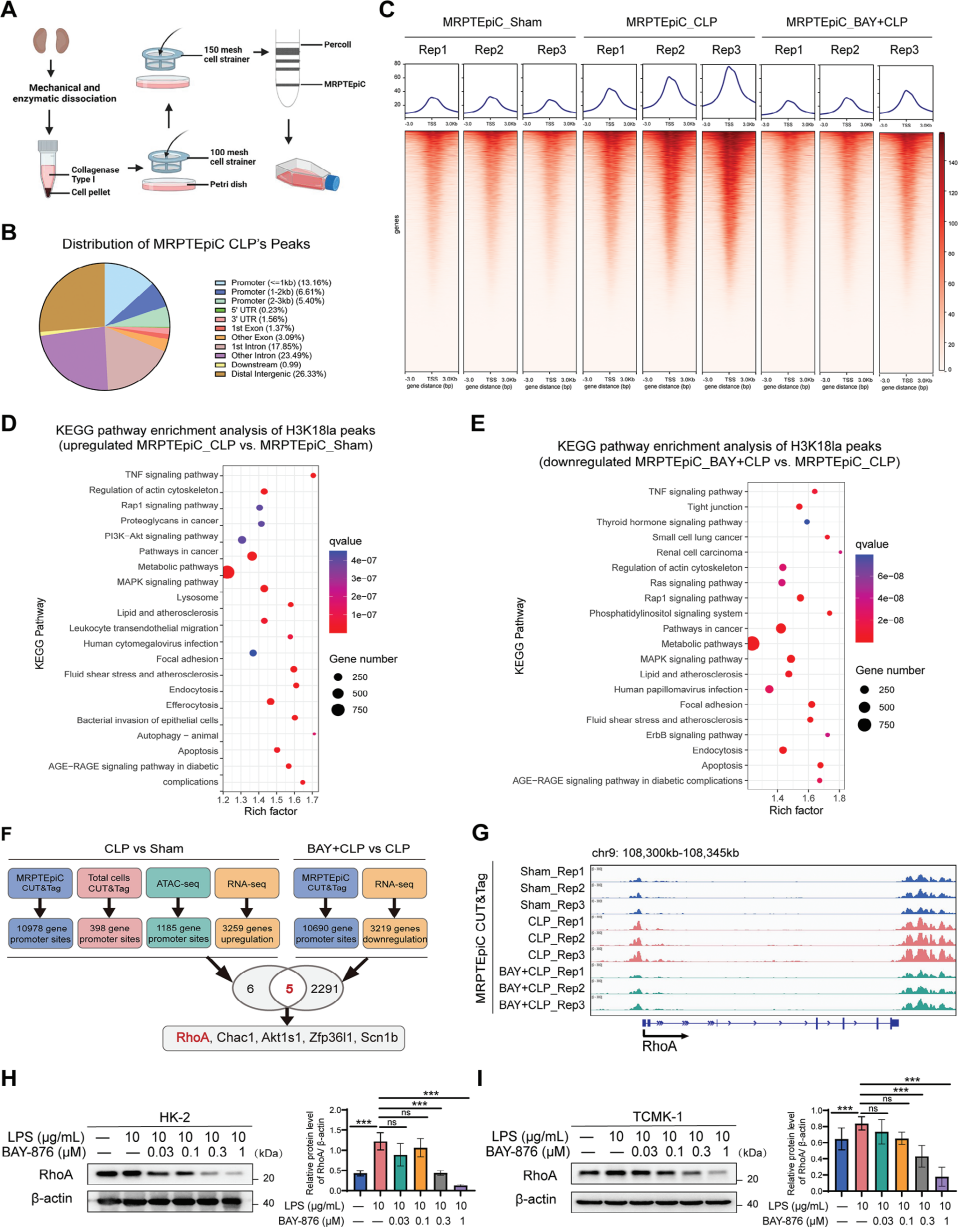
Histone lactyation activated transcription of RhoA. A) Schematic diagram of the extraction and purification of RTECs from kidney tissue. Created with BioRender.com with permission. B) Pie plot of the genomic distribution of H3K18la peaks in MRPTEpiC_CLP group. C) Heatmaps of H318la CUT&Tag signals were visualized by deepTools in MRPTEpiC_Sham, MRPTEpiC_CLP, and MRPTEpiC_BAY+CLP groups. The findings are ordered by signal strength. D) Bubble chart showing the KEGG pathway enrichment analysis of MRPTEpiC_CLP group upregulated genes with increased H3K18la modification. E) The KEGG pathway enrichment analysis of MRPTEpiC_BAY+CLP group downregulated genes with decreased H3K18la modification. F) The flow chart depecting selection of RhoA as the downstream target of H3K18la by bioinformatics analysis. G) IGV tracks for RhoA from MRPTEpiC CUT&Tag analysis. H,I) Western blot was performed to test RhoA expression in HK‐2 and TCMK‐1 cells after treatment with different concentrations of BAY‐876 and 10 µg/mL of LPS. n  =  3 independent experiments. ^*^ *p* <0.05, ^**^ *p* <0.01, and ^***^ *p* <0.001; ns, not significant.

Histone modification can affect the transcription level of target genes.^[^ ^24^, ^25^, ^26^ ^]^ Zhang et al. further demonstrated that lactate‐derived histone lactylation (for example, at the H3K18la site) can directly stimulate gene transcription.^[^ ^23^ ^]^ In order to reveal the regulatory role of histone lactylation in gene expression, we first performed ATAC‐seq analysis and CUT&Tag analysis with the whole kidney tissue of the SA‐AKI mouse model, followed by sequencing using anti‐H3K18la antibodies. The results showed that compared with the sham group, chromatin accessibility was higher in the CLP group. In CUT&Tag deep Tools analysis, the peak of H3K18la was enriched in the CLP group (Figure S3A, Supporting Information). To explore potential candidate genes regulated by H3K18la in renal tubular epithelial cells (MRPTEpiC) of SA‐AKI mice, we isolated and identified primary MRPTEpiC (**Figure** **3**A; Figure S3B–D, Supporting Information) for three independent replicate of CUT&Tag experiments (grouped as MRPTEpiC_Sham, MRPTEpiC_CLP, and MRPTEpiC_BAY+CLP). The results showed that compared to MRPTEpiC_Sham, MRPTEpiC_CLP exhibits enrichment of H3K18la peaks, with 10 978 (25.08%) H3K18la binding peaks located within promoter region (≤3Kb) (Figure 3B). After the addition of the BAY‐876 inhibitor, the enrichment of H3K18la peaks decreased compared to MRPTEpiC_CLP (Figure 3C). To investigate the epigenetic regulatory role of H3K18la in MRPTEpiC, Kyoto Encyclopedia of Genes and Genomes (KEGG) analysis was performed on the target genes of H3K18la binding peaks. The results indicate that, compared to MRPTEpiC_Sham, the H3K18la‐specific genes in the MRPTEpiC_CLP group are significantly enriched in the regulation of actin cytoskeleton. Additionally, pathways involved in metabolism and inflammation of epithelial cell, as well as cell apoptosis, are also enriched (Figure 3D). Furthermore, these enrichments can be suppressed by BAY‐876 (Figure 3E).

Next, we performed the CUT&Tag and ATAC‐seq and combined these results with those of the transcriptomic sequencing analyses of mouse kidney tissues with or without BAY‐876. RhoA, Chac1, Akt1s1, Zfp36l1, and Scn1b were selected as candidate genes (Figure 3F; Figure S3E, Supporting Information). Among these candidate genes, RhoA, a member of the small GTPase family, is critical in the regulation of cellular barrier function in a variety of inflammatory diseases such as sepsis,^[^ ^27^, ^28^ ^]^ and also plays a crucial role in cell morphology, motility, adhesion, and proliferation.^[^ ^29^, ^30^ ^]^ Hence, we aimed to focus on RhoA. To verify whether transcription of RhoA is positively correlated to H3K18la, we initially compared the signals of MRPTEpiC CUT&Tag peaks in the RhoA region. We found a significant enrichment of the H3K18la signal at the RhoA promoter in the genomic position of the CLP group compared to that in the Sham group (Figure 3G). This marked enrichment was reduced by BAY‐876 (Figure 3G). In addition, as expected, BAY‐876 treatment led to a significant decrease in protein levels of RhoA expression (Figure 3H,I).

### 2.4 LPS‐Mediated RhoA‐ROCK Pathway Induced Ezrin Phosphorylation and Sublocalization

**Figure 4:**
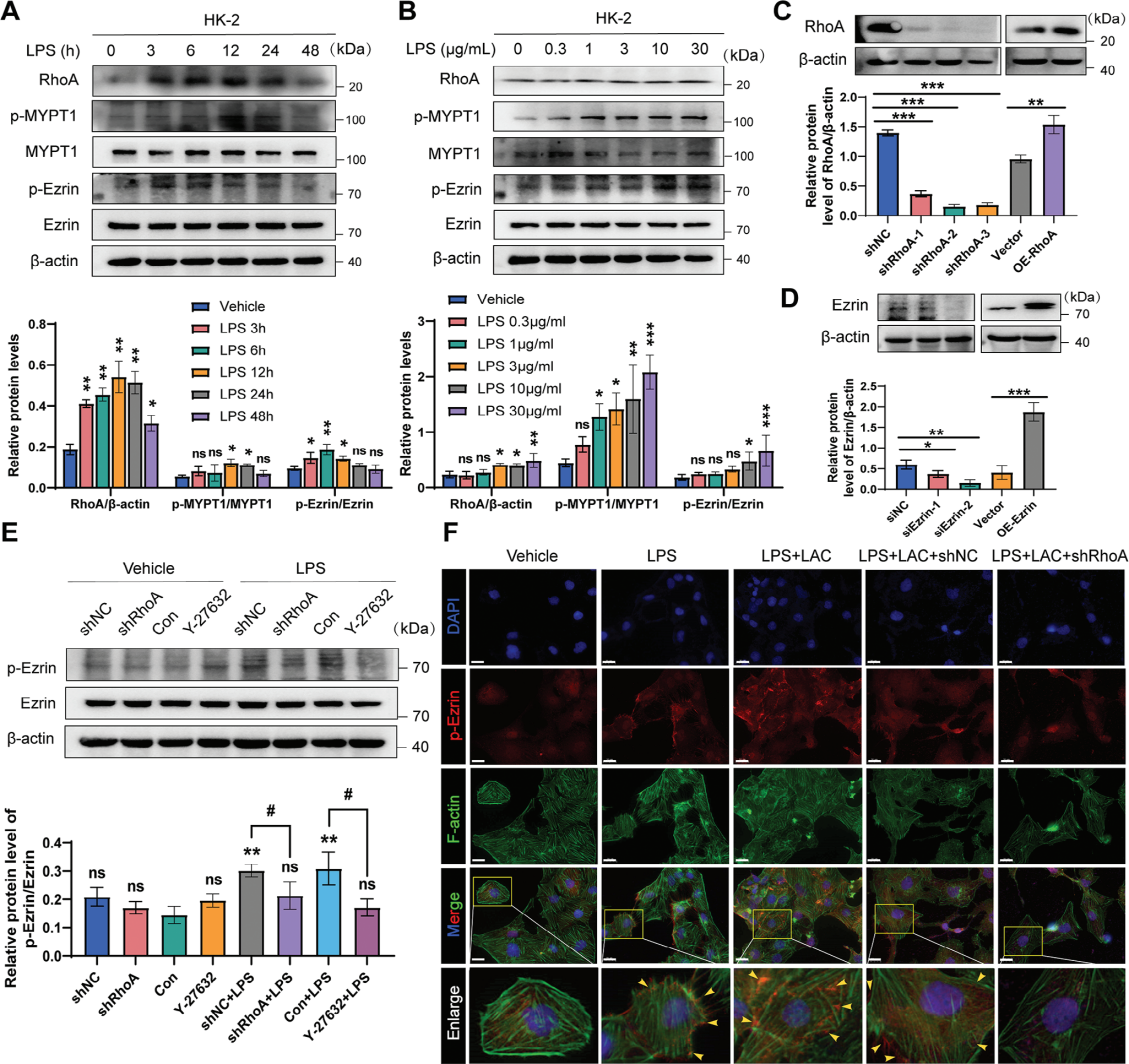
RhoA/ROCK signaling pathway mediated LPS‐induced Ezrin phosphorylation and translocation. A) HK‐2 cells were treated with LPS (10 µg mL^−1^) for 0, 3, 6, 12, 24, and 24 h, respectively. RhoA, phosphorylated MYPT1, MYPT1, phosphorylated Ezrin, and Ezrin protein level was evaluated by western blotting. B) HK‐2 cells were treated with LPS for 24 h at concentrations of 0, 0.3, 1, 3, and 10 µg mL^−1^, respectively. RhoA, MYPT1, Ezrin, phosphorylated MYPT1, and Ezrin protein level was evaluated by western blotting. C) Western blot showed that RhoA was knocked down after shRNA transfection and overexpressed after being transfected with a plasmid carrying *RhoA* gene in HK‐2 cells. D) Western blot showed that Ezrin was knocked down after siRNA transfection and overexpressed after transfected with a lentiviral vector carrying the Ezrin‐encoding gene in HK‐2 cells. n  =  3 independent experiments. ^*^ *p* <0.05, ^**^ *p* <0.01, and ^***^ *p* <0.001; ns, not significant. E) Total Ezrin and phosphorylated Ezrin protein levels were detected in HK‐2 cells challenged with RhoA shRNA or Y‐27632 ± 10 µg mL^−1^ LPS for 24 h by western blotting. ^**^ *p* <0.01 versus Con, ^#^ *p* <0.05 versus LPS. F) The localization of Ezrin was analyzed by immunofluorescence staining (scale bar = 20 µm). p‐Ezrin was stained with Alexa Fluor 594‐conjugated IgG (red), F‐actin was stained with phalloidin (green), and nuclei were stained with DAPI (blue). n  =  3 independent experiments.

To investigate the potential involvement of RhoA/ROCK signaling in LPS‐stimulated HK‐2 cells, we used western blotting to examine the expression and phosphorylation levels of myosin phosphatase target subunit 1 (MYPT1), the downstream target of RhoA and ROCK. The results of time response showed that LPS‐induced RhoA and ROCK1 protein levels increased 3 h after LPS stimulation, reached a peak at 6 h, and returned to baseline 48 h later. The dose‐response results showed a dose‐dependent increase (**Figure** **4**A,B). Notably, ROCK is involved in Ezrin activation.^[^ ^31^, ^32^, ^33^ ^]^ Therefore, we first verified the knockdown and overexpression efficiency of RhoA and Ezrin. Western blot analysis showed that transfection of RhoA shRNA and Ezrin siRNA significantly reduced RhoA and Ezrin protein expression levels. Anterograde RhoA plasmid and Ezrin lentivirus significantly increased the respective expression levels of the two proteins (Figure 4C,D). HK‐2 cells were transfected with RhoA shRNA or pretreated with ROCK1 inhibitor Y‐27632 before LPS stimulation. Western blot results showed that the protein expression level of p‐ezrin increased significantly in LPS‐treated cells. Up‐regulation of p‐Ezrin was significantly inhibited by transfection with RhoA shRNA or pretreatment with Y‐27632 (Figure 4E). In addition, immunofluorescence analysis showed the intracellular sublocalization of Ezrin. Resting cells fluoresce weakly with p‐Ezrin and are mainly located in the cytoplasm, where p‐Ezrin appears to be transferred from the cytoplasm to the membrane in response to LPS, as shown by the yellow arrow. This was further demonstrated by co‐localization with F‐actin cytoskeletal recombination. This migration was significantly restored by transfection with RhoA shRNA (Figure 4F).

### 2.5 RhoA‐ROCK1‐Ezrin Pathway Induces NF‐κB Activation, and Ezrin Inhibition Reduces the Membrane Expression of GLUT1

**Figure 5:**
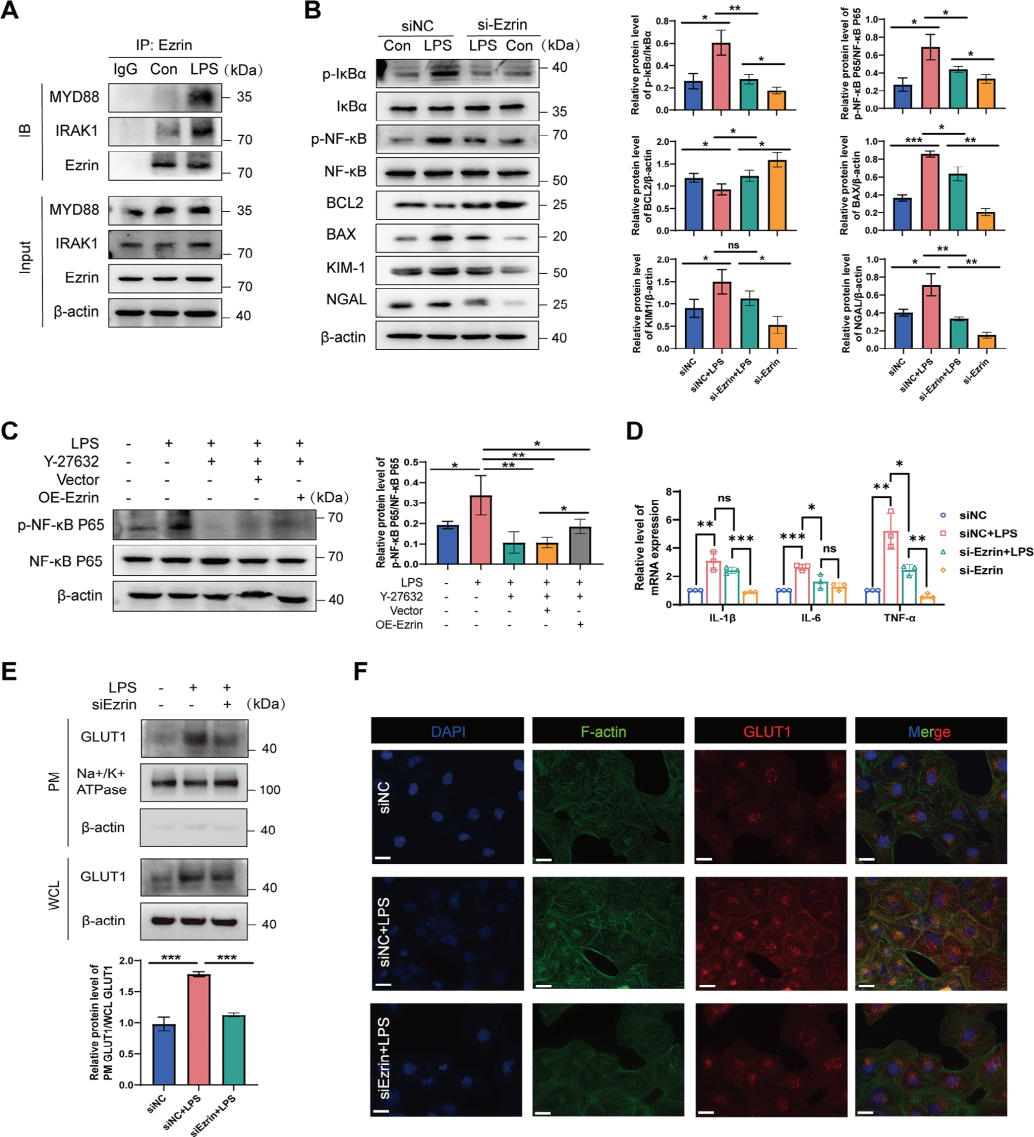
Ezrin is a key downstream gene of RhoA, regulating RTEC damage and translocation of GLUT1 in the plasma membrane. A) The association between Ezrin and MyD88/IRAK1 in HK2 cells in the presence or absence of LPS (10 µg mL^−1^) was detected by co‐immunoprecipitation. B) HK‐2 cells transfected with siNC or siEzrin and incubated with ±10 µg mL^−1^ LPS for 24 h. Western blot was performed to analyze p‐IKBα, IKBα, p‐NF‐κB, NF‐κB, BCL2, BAX, KIM‐1, and NGAL protein levels. C) The rescue experiment was performed to confirm the relationship between ROCK and Ezrin in vitro. D) Detection of IL‐1, IL‐6, and TNF‐α mRNA expression levels by real‐time qPCR, n  =  3 independent experiments. E) Western blot analysis was performed to detect the plasma membrane (PM)/ WCL whole‐cell lysate (WCL) expression level of GLUT1 in HK‐2 cells; the internal reference for cell membrane protein was Na+/K+ ATPase. n  =  3 independent experiments. ^*^ *p* <0.05, ^**^ *p* <0.01, and ^***^ *p* <0.001; ns, not significant. F) The localization of GLUT1 was analyzed by immunofluorescence staining (scale bar = 20 µm). Ezrin was stained with Alexa Fluor 594‐conjugated IgG (red), GLUT1 was stained with Alexa Fluor 488‐conjugated IgG (green), and nuclei were stained with DAPI (blue), n  =  3 independent experiments.

We examined potential interactions between Ezrin and myeloid differentiation factor 88 (MyD88)/IL‐1R‐associated kinase 1 (IRAK‐1). The interaction was significantly enhanced after LPS stimulation via COIP experiments, indicating that Ezrin binds to MyD88/IRAK1 in an LPS stimulation‐dependent manner (**Figure** **5**A). To explore further Ezrin‐mediated downstream signaling, HK‐2 cells were transfected with siNC or siEzrin in the presence or absence of LPS. Western blotting analysis was used to measure the protein expression and phosphorylation levels of NF‐κB inhibitor alpha (IκBα) and NF‐κB, as well as changes in apoptotic and renal injury biomarkers (KIM‐1 and NGAL). LPS treatment significantly increased the activation of NF‐κB in HK‐2 cells transfected with siNC. The expression level of B‐cell lymphoma protein 2 (BCL2) decreased, whereas the expression level of BAX, KIM‐1, and NGAL protein increased. Ezrin knockdown partially suppressed the increase of LPS‐induced NF‐κB activation, apoptosis, and the elevation of kidney injury markers (Figure 5B). Rescue tests further demonstrated that knockdown of RhoA and overexpression of Ezrin could restore downstream NF‐κB activation (Figure 5C). The effects of Ezrin knockdown on the proinflammatory cytokines production in HK‐2 cells with or without LPS were measured by real‐time qPCR. As anticipated, LPS mediated a significant increase in mRNA expression levels of IL‐1β, IL‐6, and TNF‐α. Knockdown of Ezrin inhibited this upregulation (Figure 5D).

Ezrin positively correlates with the expression of HK2, a key enzyme involved in glycolysis. We aimed to examine whether Ezrin was related to the sublocalization and expression of GLUT1. Total proteins were extracted from HK‐2 cells, and membrane proteins were separated using the Membrane/Cytosol/Nuclear Protein Extraction Kit (Beijing Solaibio Technology Co., Ltd., EX1400). Western blot analysis demonstrated that in HK‐2 cells, the knockdown of Ezrin resulted in a decrease in the ratio of cell membrane protein GLUT1 to total cellular protein GLUT1 (Figure 5E). This phenomenon was also confirmed by immunofluorescence (Figure 5F). The results indicate that LPS promotes GLUT1 membrane localization, while knockdown of Ezrin significantly reduces the protein levels of GLUT1 in the PM/WCL.

### 2.6 The Reduction of H3K18la has Anti‐Inflammatory Effects and Contributes to the Improvement of Renal Function After SA‐AKI

**Figure 6:**
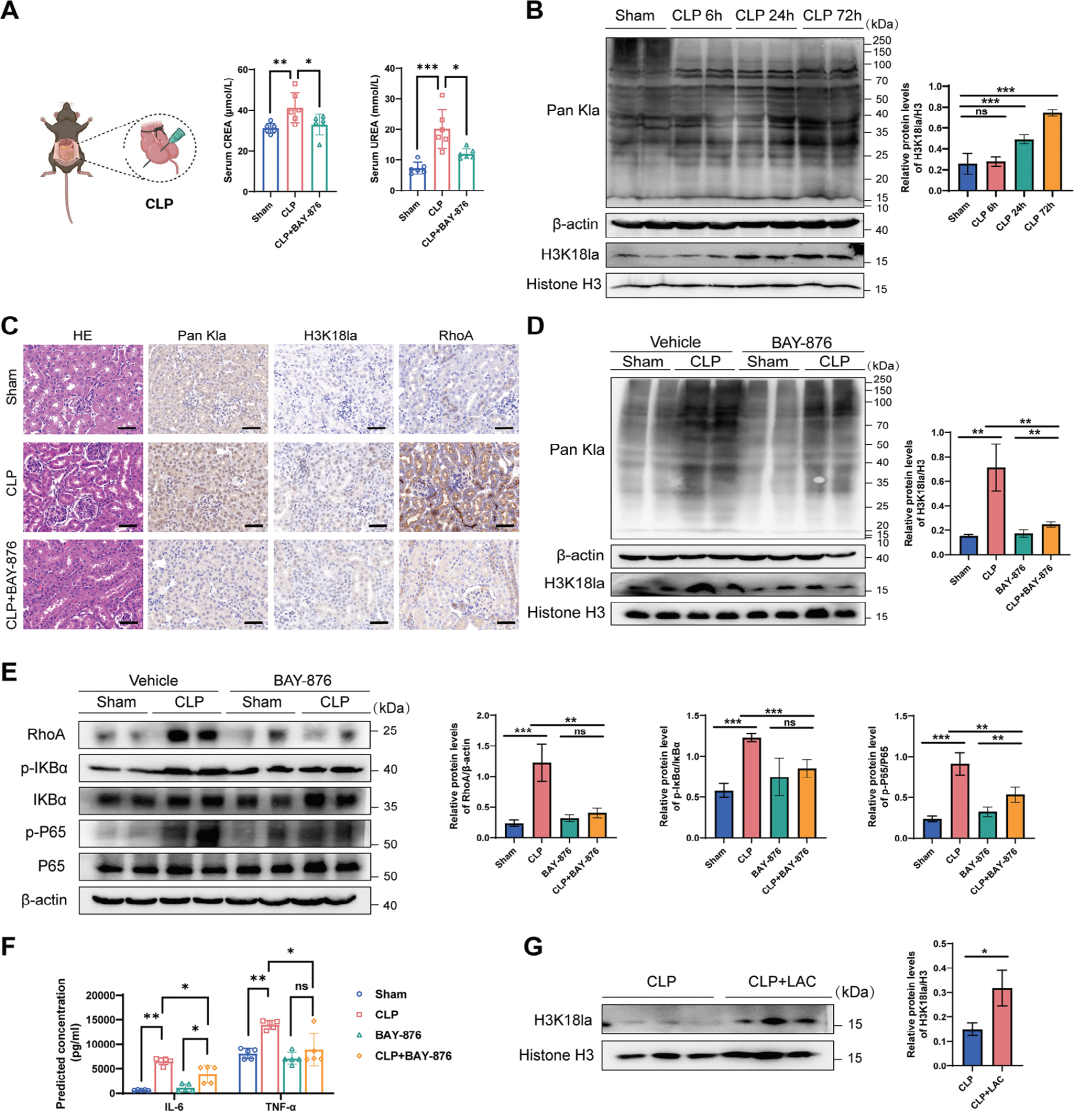
GLUT1 inhibition reduces H3K18la and improves renal function impairment after SA‐AKI. A) Mouse CLP model diagram (Created with BioRender.com with permission) and mouse serum CREA and UREA levels in the Sham, CLP, and CLP+BAY‐876 groups. B) Western blotting analysis of Pan Kla and H3K18la in the total proteins extracted from the kidney tissues of the Sham group and 6 h, 24 h, 72 h after CLP (n  = 4 mice per group). C) Hematoxylin‐eosin staining (H&E) staining showing the degree of inflammatory cell infiltration. Immunohistochemical (IHC) staining showed the protein expression levels and sublocalization of Pan Kla, H3K18la, and RhoA in mouse kidneys (scale bar = 50 µm, n  = 6 mice per group). D) Western blotting analysis of Pan Kla and H3K18la in the total proteins extracted from the kidney tissues of the Sham group and CLP group ± BAY‐876 (n  = 4 mice per group). (E) Kidney tissue protein from Sham group and CLP group ±10 µg mL^−1^ LPS for 24 h was evaluated by western blotting for the RhoA, p‐IKBα, IKBα, p‐NF‐κB, and NF‐κB protein levels. F) Serum cytokine concentrations were measured using a Mouse Inflammation Panel (13‐plex) with a V‐bottom Plate and demonstrated IL‐6 and TNF‐α levels (n  = 5 mice per group). G) Western blotting analysis of H3K18la in the total kidney tissue protein of the CLP group and CLP+LAC group (n  = 3 mice per group). ^*^ *p* <0.05 and ^**^ *p* <0.01 versus LPS group; ns, not significant versus LPS group.

Clinical evidence suggests that high serum lactate levels are associated with activity and mortality in patients with sepsis.^[^ ^34^, ^35^, ^36^ ^]^ We established a mouse model of SA‐AKI using cecal puncture ligation and observed a significant increase in serum lactate levels and serum creatinine and urate nitrogen levels (Figures 1A and 6A). In order to investigate the changes in histone lactylation at different time points of CLP, we performed western blot analysis of total protein collected from kidney tissue of mice with sepsis at different time points. The results showed that lactylation level was significantly up‐regulated 24 h after CLP and remained high until 72 h. The lactylation trend of H3K18la was consistent with the change in total lactylation level (**Figure** **6**B). Similarly, immunohistochemistry at 24 h after CLP showed a significant increase in renal H3K18la levels, and the images showed that lactylation was mainly observed in the nucleus of renal proximal convoluted tubule epithelial cells (Figure 6C).

To evaluate the effect of histone lactylation on renal function in SA‐AKI models, BAY‐876 was intraperitoneally injected 24 h before surgery to control histone lactylation in proximal tubule epithelial cells. BAY‐876 treatment inhibited renal global lactylation and H3K18la levels, decreased RhoA protein expression and downstream IKBα and NF‐κB phosphorylation levels, and inhibited the expression of inflammatory factors IL‐6 and TNF‐α (Figure 6D,E,F). Hematoxylin and eosin staining and immunohistochemical staining confirmed that H3K18la and inflammatory cell infiltration were decreased in proximal convoluted renal tubule epithelial cells compared to those in the CLP group (Figure 6C). To determine whether increased lactate levels could modulate H3K18la levels during sepsis, we increased serum lactate levels via peritoneal injection of lactate 6 h after induced sepsis and examined changes in H3K18la levels. In contrast to BAY‐876, the H3K18la levels were significantly increased after lactic acid treatment (Figure 6G).

Taken together, these results suggest that during SA‐AKI, increased H3K18la levels increase NF‐κB pathway activation and aggravate kidney injury, whereas a reduction in H3K18la has anti‐inflammatory effects with mitigation of kidney injury, contributing to the improvement of renal function after SA‐AKI.

### 2.7 Lactate Promotes Ezrin K263 Lactylation, and K263R Mutation Reverses the Regulation of Ezrin‐Mediated Kidney Injury by Lactate

**Figure 7:**
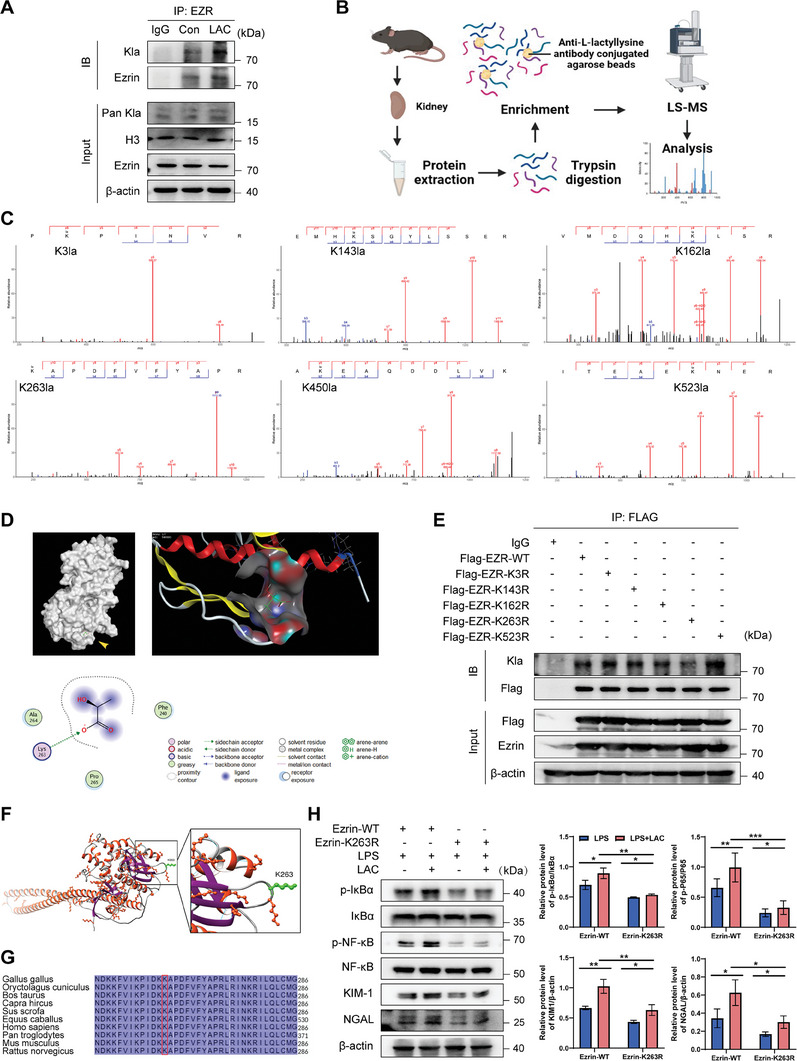
Lactate promotes K263 lactylation of Ezrin. A) Lactylation of Ezrin in HK‐2 cells was confirmed by the IP method. HK‐2 cells were treated with ddH2O or 30 mM L‐lactic acid for 24 h, followed by western blotting. B) Schematic diagram showing 4D labeled unlactated quantitative proteomics. Created with BioRender.com with permission. C) Illustration of possible lactylation sites of Ezrin in the CLP kidney tissue analyzed via immunoprecipitation (IP)‐mass spectrometry analysis. The six possible lactylation sites of Ezrin observed via IP‐mass spectrometry are shown. D) Molecular docking in the molecular operating environment (MOE) was used to predict the binding affinity between the Ezrin FREM domain and the L‐lactic acid molecule. E) Flag‐Ezrin (WT), Flag‐Ezrin (K3R), Flag‐Ezrin (K143R), Flag‐Ezrin (K162R), Flag‐Ezrin (K263R), and Flag‐Ezrin (K523R) were transfected into 293T cells for 48 h via overexpression plasmid. Proteins obtained from the Flag‐Ezrin overexpressed 293T cells were pulled down by flag antibody and detected with anti‐lactyllysine antibody. F) A ribbon diagram of the crystal structure of human Ezrin protein (P26040) was obtained from AlphaFold Protein Structure Database (https://alphafold.ebi.ac.uk). We used Chimera1.17,1 software to visualize the protein structure and label the K263 site. G) The sequences around Ezrin K263 from different mammal species were conserved. Ezrin K263 conservative lysine residues are marked in red. H) Ezrin‐WT or Ezrin‐K263R site mutation in overexpressed HK‐2 cells was constructed via the lentiviral vector. Then, the cells were incubated with 10 µg/mL LPS ± 30 mm lactate for 24 h. Cell proteins were analyzed by WB for p‐IKBα, IKBα, p‐NF‐κB, NF‐κB, KIM‐1, and NGAL protein levels. n  =  3 independent experiments. ^*^ *p* <0.05 and ^**^ *p* <0.01; ns, not significant.

CUT&Tag sequencing using anti‐H3K18la antibodies was performed in whole kidney tissue of SA‐AKI mouse models. DeepTools analysis showed that H3K18la peaks were significantly enriched in the CLP+lactate (CLP+LAC) group compared to those in the CLP group (Figure S4A, Supporting Information). Genome Explorer tracked CUT&Tag signals at the representative target locus Ezrin, and these peaks identified elevated H3K18la levels in the Ezrin gene promoter region. Moreover, no significant change was observed in the chromatin accessibility of the Ezrin gene in ATAC‐seq (Figure S4B, Supporting Information). To determine whether Ezrin can be lactated in cells, we transfected Ezrin‐overexpressing lentivirus into HK‐2 cells and added 30 mm lactate to the medium. Immunoprecipitation tests were then performed, Ezrin was pulled down with anti‐Ezrin antibodies, and Ezrin lactatation levels were detected with anti‐acetylation antibodies. We observed that Kla was indeed present on Ezrin, and the presence was significantly enhanced after lactic acid treatment (**Figure** **7**A). These results indicate that *e*xogenous lactate supplementation promoted an increase in Ezrin lactylation levels.

PTMs plays a central role in the functional regulation of proteins, and lactic acid can directly modify proteins.^[^ ^23^ ^]^ The regulatory mechanism by which the lactated modification of Ezrin influences its function remains elusive. Next, we explored the underlying mechanism further. The kidney tissues of mice in the Sham group and CLP group were subjected to 4D label‐free quantitative proteomics with lactylation modification‐specific proteomics analysis (Figure 7B). No significant difference was observed in Ezrin protein levels in the proteomics analysis. However, liquid chromatography‐mass spectrometry (LC‐MS) analysis showed that there may be significant differences in lactylation sites K3, K143, K162, K263, K450, and K523 in Ezrin (Figure 7C). We used molecular docking in the molecular operating environment (MOE) to predict the binding affinity between L‐lactic acid molecules and the Ezrin FERM domain, predicting the site where the amino acid will be lactated (Figure 7D). It is generally believed that the interaction of the lysine residue K253/254/262/263 in the ezrin FERM domain with phosphatidylinositol, 4, 5‐diphosphate (PIP2) is essential for activation.^[^ ^37^, ^38^ ^]^ In this study, we mutated lysine to arginine for de‐lactate modification. By overexpressing Flag‐Ezrin‐WT plasmid into 293T cells, we constructed 293T cells mutated at K3R, K143R, K162R, and K523R due to poor species‐specific specificity of K450 sites. The results showed that the level of Flag in Ezrin overexpressed cells was significantly up‐regulated. Next, we pulled down Ezrin with Flag antibodies and detected altered lactate levels of Ezrin point mutations using anti‐lactylation antibodies. We found that mutations at K263R significantly reduced Ezrin lactylation, whereas mutations at other sites had no significant effect on lactylation levels (Figure 7E). The tertiary structure of Ezrin indicated that K263 was located in the FERM domain of the Ezrin protein (Figure 7F) and was conserved in a variety of mammalian species (Figure 7G). These results indicate that K263 is the leading lactylation site of Ezrin, and Anti‐Lactyla‐Ezrin (K263) antibody validation was performed (Figure S4C–F, Supporting Information).

Next, we attempted to investigate the regulatory role of Ezrin‐K263 lactylation on the NF‐κB pathway by overexpressing Ezrin‐WT and Ezrin‐K263R mutations in HK‐2 cells. The addition of lactate increased NF‐κB activation levels and cell damage in HK‐2 cells to those observed in the Ezrin‐WT+LPS group. However, this effect was significantly diminished in K263R mutated cells. Compared with the Ezrin‐WT+LPS+LAC group, the Ezrin‐K263R+LPS+LAC group also showed lower levels of NF‐kB phosphorylation and alleviated cell damage (Figure 7H). These results support that lactate promoted LPS‐induced renal injury exacerbation via lactylation of Ezrin at K263, which may be a potential lactylation modification target.

## 3 Discussion

Despite significant advances in understanding the pathophysiology and detection markers of SA‐AKI, it remains a common and hazardous complication of critical illness.^[^ ^39^ ^]^ The pathophysiology of SA‐AKI involves damage and dysfunction in many cell types, including macrophages, vascular endothelial cells (ECs), and RTECs, as well as their crosstalk and associations.^[^ ^40^ ^]^ Accumulating evidence suggests that the pathogenesis of SA‐AKI is multifactorial and complex, involving interactions between inflammation, microcirculatory dysfunction, and metabolic reprogramming. However, the mechanisms in terms of epigenetic regulation are not fully understood.^[^ ^41^ ^]^ This study investigated the changes in lactylation modifications in SA‐AKI and explored their relationship with glycolysis inhibitors. Despite the stability of global histone acetylation and H3K18ac, global histone lactylation and H3K18la showed a significant increase in SA‐AKI, which was markedly reduced upon the addition of glycolysis inhibitors. In previous studies, hypoxia‐induced lactate production in MCF‐7 cells increased the level of histone lactylation but did not elevate the level of histone acetylation.^[^ ^23^ ^]^ Moreover, hypoxia did not significantly alter the acetylation levels of H3K23ac and H3K18ac before embryo implantation.^[^ ^42^ ^]^ H3K18la and H3K18ac levels do not follow the same temporal dynamics. In liver stellate cell activation and hepatic fibrosis, despite an increase in lactate and acetyl‐CoA levels induced by the upregulation of glycolysis through HK2 expression, the impact was limited to histone lactylation and did not induce histone acetylation.^[^ ^43^ ^]^ Therefore, these studies suggest functional differences in the patterns of histone lactylation and acetylation.

RhoA is a universally expressed cytoplasmic protein belonging to the small GTPase family that acts as a molecular switch, is activated in response to the binding of chemokines, cytokines, and growth factors, and can regulate the activation of cytoskeletal proteins and other factors via a ROCK signaling cascade.^[^ ^44^ ^]^ The RhoA/ROCK pathway can promote the progression of various diseases, such as inflammatory bowel disease,^[^ ^29^ ^]^ those involving microglia activation,^[^ ^45^, ^46^ ^]^ and acute lung injury.^[^ ^47^, ^48^ ^]^ In a rat model of unilateral ureteral obstruction (UUO), extracellular vesicles produced by bone marrow mesenchymal stem cells mitigated renal fibrosis by inhibiting the RhoA/ROCK pathway.^[^ ^49^ ^]^ The role of RhoA in SA‐AKI remains to be explored. This study demonstrated that the expression level of RhoA protein and phosphorylation level of ROCK1 downstream target protein MYPT1 increased in SA‐AKI, and the inhibition of GLUT1 decreased the expression of RhoA protein.

The Ezrin/radixin/moesin (ERM) protein family consists of an N‐terminal FERM domain (a dense clover‐shaped structure consisting of three structural modules F1, F2, and F3) and a C‐terminal domain containing an F‐actin binding site, connected by a central alpha helix structure.^[^ ^50^, ^51^ ^]^ The FERM domain is involved in binding membrane proteins and scaffold proteins.^[^ ^52^, ^53^ ^]^ Ezrin is a member of the ERM protein family whose n‐terminal FERM domain interacts with the C‐terminal domain when dormant, whereas PtdIns (4,5) P_2_ binds to the F3 lobe of the FERM domain. Subsequently, phosphorylation of the C‐terminal domain Thr567 residues of RhoA downstream effector ROCK leads to the transition from the inactive to the active form, and Ezrin is recruited from the cytoplasm to the membrane.^[^ ^54^, ^55^ ^]^ Y‐27632 is a ROCK inhibitor that blocks Ezrin phosphorylation and binds to the actin cytoskeleton.^[^ ^56^ ^]^ Based on the evidence indicating that Y‐27632 blocks Ezrin phosphorylation, this study further explored whether Ezrin bonded with MYD‐88 and IRAK1 in a LPS‐dependent manner to induce downstream NF‐κB activation and release of inflammatory factors, leading to the aggravation of kidney injury. We found that Ezrin may be partially involved in regulating glycolytic reprogramming and GLUT1 sublocalization and expression.

PTMs of proteins include acetylation, methylation, phosphorylation, ubiquitination, and glycosylation.^[^ ^12^ ^]^ Histone acylation is a crucial component of epigenetic regulation of gene transcription, and acetylation has been the first observed and most studied in renal diseases, including ischemia‐reperfusion injury‐induced AKI‐CKD transition model and unilateral ureteral obstruction‐induced renal fibrosis model.^[^ ^57^, ^58^ ^]^ Non‐histone acylation regulates protein functions such as protein activity, stability, and interactions. For example, p53 deacetylation can reduce acute kidney injury caused by sepsis by promoting autophagy,^[^ ^59^ ^]^ and SIRT1‐mediated HMGB1 deacetylation can inhibit sepsis‐related acute kidney injury.^[^ ^60^ ^]^ Recently, novel lysine acylation reactions, including propionylation, butyrylation, malonylation, succinylation, crotonylation, 2‐hydroxy‐butyrylation, β‐hydroxy‐butyrylation, glutarylation, and lactylation, have been extensively examined. As a potential modification substrate, lactate regulates epigenetic regulation of histones by introducing lactyl functional groups to histones. including H3K9,^[^ ^61^ ^]^ H3K14,^[^ ^62^ ^]^ H3K18,^[^ ^17^, ^63^ ^]^ h3k23,^[^ ^42^ ^]^ H3K56,^[^ ^61^ ^]^ and H4K12,^[^ ^62^, ^64^, ^65^ ^]^ thereby regulating the transcription of specific genes. Subsequently, Non‐histone lactylation modifications have been reported, and they can also regulate the transcription of genes, such as those encoding HMGB,^[^ ^66^ ^]^ PKM2,^[^ ^64^ ^]^ HIF1α,^[^ ^67^ ^]^ Snail1,^[^ ^14^ ^]^ YY1,^[^ ^68^ ^]^ and METTL3.^[^ ^69^ ^]^ H3K18la is involved in the expression of homeostatic genes related to damage repair, such as Arg‐1, playing a crucial role in the mechanisms of homeostasis and healing in vivo.^[^ ^23^ ^]^ In a single‐center, small‐sample clinical study on sepsis, researchers examined peripheral blood mononuclear cells from healthy volunteers and 35 critically ill patients (septic and nonseptic). The study revealed that H3K18la may be associated with the severity of critical illness and infection. Furthermore, H3K18la showed a positive correlation with the production of inflammatory factors.^[^ ^70^ ^]^ In a study by Pan et al., enhanced H4K12 lactylation in AD model mice and microglia exacerbated the cognitive impairment phenotype via the glycolysis/H4K12la/PKM2 axis.^[^ ^64^ ^]^ In contrast, H3K18la and H4K12la exhibited different localization and biological functions with distinct downstream targets in the pathogenesis of Alzheimer's disease. Lin et al. found that aging microglia undergo a metabolic shift to aerobic glycolysis regulated by the enhanced H3K18la/NFκB axis to modulate age‐related inflammation by regulating SASP components IL‐6 and IL‐8.^[^ ^71^ ^]^

However, the changes and functions of these novel acetylations in the kidneys have not been extensively examined, especially since the role of lactylation modification in the kidneys has not been reported to date. We conducted the first exploration of the potential roles of histone lactylation in the development of SA‐AKI and generated the initial dataset of lactylation modifications on key enzymes in glycolysis and the tricarboxylic acid cycle in renal tissues of SA‐AKI (SFig 5). This dataset provides a foundation for further analysis of the physiological mechanisms underlying SA‐AKI. We observed an elevation in histone lactylation levels in SA‐AKI, at least partly mediated by histone lactylation regulating gene expression to modulate cellular metabolism, demonstrating the correction of abnormal lactate levels to effectively protect against renal function damage. Elevated histone lactylation promoted RhoA expression and induced phosphorylation and sublocation of RhoA/ROCK pathway. Notably, we demonstrated a significant increase in Ezrin lactylation after CLP We also identified multiple lactylation sites, with K263 defined as a potential lactylation target. Under in vitro conditions, an increase in lactate levels in LPS‐stimulated renal tubular epithelial cells, accumulation of endogenous lactate, and supplementation of exogenous lactate all led to an increase in Ezrin levels in renal tubular epithelial cells. This partially elucidates the association between high blood lactate levels and the high incidence and adverse prognosis of SA‐AKI. The increase in Ezrin may further promote inflammatory responses, primarily attributed to the interaction of Ezrin with MYD88 and IRAK1, thereby activating the NF‐KB pathway. Additionally, mutation of the K263 site (K263R) was found to reverse the regulatory effects of lactate on Ezrin‐mediated renal injury. The findings of this study enhance our comprehension of Ezrin's regulatory mechanisms and further elucidate the physiological significance of lactate modification in renal diseases.

Finally, in this study, SA‐AKI promoted the increase in lactic acid and H3K18la, activated the expression of RhoA protein, and mediated downstream inflammation and apoptosis, contributing to kidney injury. Conversely, inhibition of GLUT1 alleviated the increase in H3K18la, providing a new mechanistic foundation for delaying renal dysfunction. Our data suggest that glycolysis‐derived lactate and H3K18la may be potential targets for improving SA‐AKI renal function via inhibition of RhoA/ROCK/Ezrin signaling and Ezrin K263 lactylation mediated by reducing NF‐κB activation.

## 4 Experimental Section

### Antibodies and Reagents

LPS (L2630, E. coli 0111: B4) was purchased from Sigma–Aldrich (MO, United States). BAY‐876 (HY‐100017), 2‐DG (HY‐13966), L‐(+)‐lactic acid (Lactate, HY‐B2227), and Y‐27632 (HY‐10071) were obtained from MedChemExpress (Shanghai, China). Oxamic acid sodium (O7100) and Rotenone (C1866) were obtained from Lablead (Beijing, China).

Anti‐L‐Lactyl Lysine (PTM‐1401RM), Anti‐Lactyl‐Histone H4 (Lys5) Rabbit mAb (PTM‐1407RM), Anti‐Lactyl‐Histone H4 (Lys8) Rabbit mAb (PTM‐1415RM), Anti‐Lactyl‐Histone H3 (Lys9) Rabbit mAb (PTM‐1419RM), Anti‐Lactyl‐Histone H4 (Lys12) Rabbit mAb (PTM‐1411RM), Anti‐Lactyl‐Histone H3 (Lys18) Rabbit mAb (PTM‐1406 and PTM‐1427RM), Anti‐Acetyllysine Rabbit mAb (PTM‐105RM), Anti‐Acetyl‐Histone H3 (Lys18) Rabbit mAb (PTM‐114RM), and Anti‐Lactyl‐Ezrin (K263) Rabbit pAb were obtained from PTM Bio Inc. The glycolysis Antibody Sampler Kit (#8337) and the antibodies against GLUT1 (#73 015), phospho‐Ezrin (Thr567)/Radixin (Thr564)/Moesin (Thr558) (#3726), NF‐κB p65 (#8242), IκBα (#4814), Phospho‐IκBα (Ser32/36) (#9246) were obtained from Cell Signaling Technologies. Anti‐RhoA (sc‐418), anti‐MYPT1 (sc‐514261), phospho‐MYPT1 (sc‐377542), anti‐Ezrin (sc‐58758), anti‐MyD88 (sc‐74532), anti‐IRAK1 (sc‐5288), anti‐phospho‐NF‐κB p65 (Ser536) (sc‐136548), and anti‐TIM‐1 (sc‐518008) were obtained from santa cruz biotechnology. The antibody anti‐LCN2 (26991‐1‐AP) and anti‐DYKDDDDK tag (66008‐4‐Ig) were from Proteintech. Anti‐β‐Actin (A0101) was obtained from Lablead. HRP‐conjugated Goat Anti‐Rabbit IgG (H + L) secondary antibody (S0101) and Goat Anti‐Mouse IgG (H + L) secondary antibody (S0100) were purchased from Lablead. Anti‐Histone H3 antibody (ab1791), Goat Anti‐Rabbit IgG H&L (Alexa Fluor 488) (ab150077), Goat Anti‐Mouse IgG H&L (ab150080), and Goat Anti‐Mouse IgG H&L (ab150116) were obtained from ABCAM.

### Cell Lines and Cell Cultures

The HK‐2 cell line (SCSP‐511) was purchased from Cell Resource Center (IBMS, CAMS/PUMC, China), and the following cell lines were obtained from American Type Culture Collection (ATCC): TCMK‐1 (CCL‐139) and HEK293T(CRL‐11268). All cell lines used in this study were authenticated by STR profiling, and the absence of mycoplasma contamination was confirmed. The HK2 and HEK293T cells were maintained in Dulbecco's modified Eagle's medium (DMEM, Gibco, MD, United States) containing 10% heat‐inactivated fetal bovine serum (FBS, ThermoFisher, United States). TCKM‐1 cells were cultured in Minimum Essential Medium (MEM, Gibco, MD, United States) supplemented with 10% FBS. Cells were maintained in an incubator with 5% CO_2_ at 37 °C.

### Dose‐Dependent Assay

A 96‐well plate containing 100 µL of cell suspension and 2×103 cells per well was incubated in an incubator containing 5% CO2 at 37 °C for 24 h for preculture. The culture plate was treated for three days with 10 L of various drug concentrations containing varying amounts. Each well received 10 µL of CCK‐8 reagent (TargetMol, C0005) followed by incubation for 2 h in the incubator. The absorbance was calculated at 450 nm using a microplate reader.

### Oxidative Stress Markers Determination

The HK‐2 cell supernatant was collected. Markers of oxidative stress, including superoxide dismutase (SOD, S0101S) and malondialdehyde (MDA, S0131S), were measured according to the manufacturer's instructions using a test kit purchased from Beyotime Biotechnology (Shanghai, China).

### ROS Assay

The HK‐2 cells were stimulated with 10 µg mL^−1^ of LPS in a six‐well plate for 12 h. In the BAY‐876 group, cells were pretreated with BAY‐876 (30 nM) 3 h before LPS administration. The probe was then loaded on ROS Assay Kit (Beyotime Biotechnology, Shanghai, China, S0033S). DCFH‐DA was diluted in a serum‐free medium at a ratio of 1:1000 to obtain a final concentration of 10 µmol L^−1^. The cell culture medium was removed, and 1 mL of diluted DCFH‐DA was added. Subsequently, incubation was performed in a cell incubator at 37°C for 20 min. The cells were washed thrice with serum‐free cell culture solution to remove the DCFH‐DA that did not completely penetrate the cells. The cells were collected and analyzed using a fluorescent enzyme label. The excitation and emission wavelengths of 488 and 525 nm were used to detect the intensity of fluorescence.

### Apoptosis Assay

The apoptosis rate of cultured HK‐2 cells was determined using flow cytometry with Annexin V‐FITC/PI (Dojindo Laboratories, AD10). Cell preparation was conducted in accordance with the manufacturer's instructions. The cells were simply rinsed once with cold PBS and then resuspended in 100 µL of 1 binding buffer. Then, 5 µL of Annexin V was added to the cell suspension. The cells were then gently mixed and incubated for 15 min at room temperature in the dark. After adding 5 µL of PI and 400 µL of 1x binding buffer, the cells were analyzed by flow cytometry for 1 h.

### Lactic Acid Level Detection

Kidney tissue, serum, or cultured HK2 cells were homogenized with lysis buffer solution and subjected to ultrasound on ice at 300 W (on for 3s, off for 7s) for 3 min, followed by centrifugation at 4 °C at 12,000 *g* for 10 min.^[^ ^7^ ^]^ The supernatant was collected, and the lactic acid concentration was determined using the Lactic Acid assay kit (Nanjing Jiancheng Bioengineering Institute, China, A019‐2‐1) per the manufacturer's instructions.

### Western Blotting

By adding 1% protease inhibitor (Shanghai Epizyme Biomedical Technology Co., Ltd., GRF101) and 1% phosphatase inhibitor (GRF102) to RIPA lysate (Applygen Technologies Inc., C1053‐100), total cell and tissue proteins were extracted. A membrane/cytoplasmic/nucleoprotein extraction kit (Beijing Solarbio Science & Technology Co. Ltd., EX1400) was used to isolate GLUT1 membrane protein. BCA protein assay kits (Invitrogen, 23 227) were used to assess membrane and total protein concentrations. Bands from a 10% SDS‐PAGE gel were transferred to a PVDF membrane (Millipore, IPVH00010). The membranes were incubated with 5% skim milk with 0.05% Tween 20 (TBST) at room temperature for 1 h and then treated with primary antibody (1:1000 dilution) overnight at 4 °C. The membranes were rinsed thrice in TBST and then incubated with horseradish peroxidase‐coupled secondary antibody (1:5000). Finally, a 1:1 Pierce ECL western blotting substrate (Millipore, WBKLS0500) was used to reveal proteins. The detected signals were quantified using the ImageJ software and normalized based on the following internal references: β‐actin (for total protein), histone H3 (for histone proteins), or sodium‐potassium ATPase (Na+/K+ ATPase, for membrane proteins).

### Coimmunoprecipitation Assay

The HK‐2 cells were collected and washed once with pre‐cooled PBS. A total of 0.5 mL of pre‐cooled IP lysis buffer (Beyotime Biotechnology, Shanghai, China, P0013, with protease and phosphatase inhibitors) was added, followed by incubation on ice for 20 min. Subsequently, centrifugation was performed at 14 000 *g* at 4 °C for 10 min. A total of 50 µL of supernatant was obtained as the input. The remaining supernatant was transferred to a new EP tube, and 2 µg of antibody against the target protein was added. A total of 2 µL of IgG antibody was added to the IgG sample and incubated overnight at 4 °C. IP and IgG samples were supplemented with 20 µL of Agarose A+G (Santa Cruz Biotechnology, sc‐2003) and incubated via shaking in a vertical mixer at 4°C for 2 h (Agarose was precleaned with PBS thrice and rotated at 4 °C for 1 h). Subsequently, centrifugation was performed at 3000 rpm for 1 min at 4 °C to remove the supernatant. The samples were washed thrice with PBS and centrifuged at 3000 rpm for 1 min at 4 °C to remove the supernatant. Subsequently, 40 µL of IP lysis buffer and protein loading buffer were added (Shanghai Epizyme Biomedical Technology Co., Ltd, China, LT101), mixed well, and boiled for 5 min. The supernatant was extracted for subsequent experiments.

### RNA Isolation and Quantitative Real‐Time PCR (qRT‐PCR)

TRIZOL reagent (ThermoFisher, 15 596 018) was used to extract total RNA from kidney tissues and cells. RNA concentration and purity were measured by NanoDrop One Microvolume UV–vis Spectrophotometers (Thermo Scientific, 701–058112) and were transformed into cDNA using a specialized cDNA synthesis kit (Yeasen Biotechnology (Shanghai) Co., Ltd., China, ThermoFisher) according to the manufacturer's protocol. On the 7500 Real‐Time PCR system (ABI), quantitative RT‐PCR was used to assess gene expression. β‐actin was used as the internal control to normalize target gene expression. The 2^−ΔΔCT^ relative quantification method was used to calculate expression. Table S1 (Supporting Information) includes a list of the primer sequences.

### Immunofluorescence Analysis

HK‐2 cells were analyzed on a six‐well plate with a density of 2×10^5^ cells per well. The cells were fixed for 10 min with 4% paraformaldehyde, penetrated for 5 min at room temperature with 0.3% Triton X‐100, and sealed for 30 min at room temperature with 5% BSA. Subsequently, the cells were incubated with antibodies against H3K18la (1:50), GLUT1 (1:100), and p‐Ezrin (1:100) overnight. The cells were then washed and incubated for 30 min at 37°C with goat anti‐Rabbit IgG H&L (Alexa Fluor 488) (1:200). The secondary antibody was combined with the F‐actin (1:100) probe for co‐incubation. The nuclei were stained with 4′, 6‐diaminyl‐2‐phenylindoles (DAPI, Sigma‐Aldrich). Pannoramic MIDI (3D HISTECH) was used to gather images.

### RNA‐seq and analysis

RNA was extracted from mouse kidney tissues by Trizol method, and RNA‐seq libraries were generated using the NEBNextUltra RNA Library Prep Kit for Illumina (NEB, #E7530). All experiments were repeated thrice. All samples were sequenced using Illumina novaseq 6000 platform and PE150 sequencing strategy. After the CASAVA base identification was complete, the sequence data was converted to fastq format. FastQC v0.11.9 and Trimmomatic v0.39 were used to perform quality control and adapter clipping. Clean fastq data was aligned against mm10 mouse genome by STAR v2.7.10a and SAMtools v1.16.1. R v4.2.1 and DEseq2 v1.20.0 were used for differential expression analysis. Gene Ontology analysis (GOTERM_BP_DIRECT) and Pathways analysis (KEGG_Pathway) were carried out using DAVID Bioinformatics 2021.

### CUT&Tag

Briefly, MRPTEpiC cells were collected and bound to a bean protein A‐coated bead. According to the kit instructions, the cells were incubated with ConA beads, primary antibody (anti‐H3K18la antibody), secondary antibody, and hyperactive pA/G‐Tnp transposons and then fragmented. DNA fragments were extracted from the samples, amplified by PCR, and the products were purified. The CUT&Tag Library was constructed using the Hyperactive Universal CUT&Tag Assay Kit for Illumina Pro (Vazyme, China, #TD904) and sequenced on the Illumina novaseq 6000 platform.

Bioinformatic analysis of CUT&Tag data: Following the tutorial from Steven Henikoff's laboratory, FastQC v0.11.9 was employed for quality control of the raw sequencing reads. Trimming of low‐quality bases and adapter sequences from the raw reads was performed using Trimmomatic v0.39. The filtered reads were aligned to the reference mouse genome assembly mm10 using Bowtie2 v2.4.4. The SAMtools v1.9 was employed for sorting, and the sorted bam file was converted to a bedgraph file using the *genomecov* function of Bedtools v2.30.0. SEACR (specifically developed for CUT&RUN and applicable to low‐background chromatin analysis data such as CUT&Tag) v1.3 was employed to perform peak calling. Differential analysis of CUT&Tag peaks was conducted by using DESeq2 v1.36.1. Visualization of called peaks was conducted using IGV v2.14.1.

### ATAC‐Seq

According to the manufacturer's instructions, whole mouse kidney tissue was prepared using a nuclear extraction kit (Solarbio, China, SN0020). The procedure involved incorporating 5 mm sodium butyrate (pH 8.0, Beyotime Biotechnology, S1539) and 1×protease inhibitor (Shanghai Epizyme Biomedical Technology Co., Ltd., GRF101). The nucleus extracted from 1/20 whole kidney tissues was washed once with PBS and then re‐suspended with cold lysis buffer (10 mm Tris‐HCl, pH 7.4, 10 mm NaCl, 3 mm MgCl2, 0.1% NP‐40).^[^ ^72^ ^]^ The nucleosome was interrupted by the Tn5 translocation enzyme (Vazyme, China, #TD501). Purified DNA was obtained by purifying the fragment product, amplified, and barcoded with TruePrepTM Index Kit V2 for Illumina (Vazyme, China, #TD202). The amplified products were sorted by length using VAHTSTM DNA Clean Beads, and the library quality was analyzed by BioAnalyzer 2100. Sequencing was performed using the Illumina novaseq 6000 platform.

### The Proteomics and 4D Label‐Free Lactylation Quantitative Proteomics

For the proteomics and 4D label‐free lactylation quantitative proteomics, whole protein from the renal tissues of mice in the sham group and CLP group was used. The analyses were performed by PTM Bio (China). The screening of the differentially modified protein sites followed the following criteria: 1/1.5‐fold change threshold. FDR was adjusted to <1%.

### siRNA, shRNA, and Plasmid Transfection

We purchased Ezrin siRNA (siEzrin‐1, siEzrin‐2), GLUT1 siRNA (siGLUT1), LDHA siRNA (siLDHA), and LDHB siRNA (siLDHB) from Shanghai GenePharma Co., Ltd., China. The sequences were listed as follows: siEzrin‐1, 5′‐GUGAAGGAAGGAAUCCUUATT‐3′; siEzrin‐2, 5′‐GGGCAACCAUGAGUUGUAUTT‐3′; siGLUT1, 5′‐GGAAUUCAAUGCUGAUGAUTT‐3′; siLDHA, 5′‐CAACUGCUGUCACCUUCUATT‐3′; siLDHB, 5′‐GAAAUGUCAACGUGUUCAATT‐3′. According to the manufacturer's protocol, jetPRIME in vitro DNA&siRNA transfection reagent (Polyplus) was added, and HK‐2 cells were transfected with 50 nM siRNA. The human RhoA lentivirus plasmid was obtained from Mailgene biosciences Co. Ltd., China, and the sequence was as follows: RhoA shRNA‐1 5′‐CGGCCCAGACTAGATGTAGTATTTCTCGAGAAATACTACATCTAGTCTGGGTTTTTG‐3′; RhoA shRNA‐2 5′‐CCGGGAAAGACATGCTTGCTCATAGCTCGAGCTATGAGCAAGCATGTCTTTCTTTTTG‐3′; RhoA shRNA‐3 5′‐CCGGGCTCATAGTCTTCAGCAAGGACTCGAGTCCTTGCTGAAGACTATGAGCTTTTTG‐3′. The lentivirus was produced in a 293T package and infected with HK‐2 cells, followed by the addition of 5 µg mL^−1^ polybrene. Cells were selected with 1 µg mL^−1^ purinomycin 24 h after lentivirus infection and tested in vitro 5 days later. Overexpressed plasmids pCMV‐MCS‐3Flag‐EZR and pCMV‐MCS‐3Flag‐RhoA (Mailgene biosciences co. ltd., China, NM_0 03379.4, NM_0 01664.3) were constructed using the cDNA of Ezrin and RhoA and transfected with the corresponding empty vector into HK‐2 cells using jetPRIME in vitro DNA&siRNA transfection reagent according to the manufacturer's instructions.

### Site‐Directed Mutagenesis of Lysine and Transfection

Human wild type (WT) or K3R mutant Ezrin (Ezrin‐K3R), K143R mutant Ezrin (Ezrin‐K143R), K162R mutant Ezrin (Ezrin‐K162R), K263R mutant Ezrin (Ezrin‐K263r), and K523R mutant Ezrin (Ezrin‐K523r) (3‐flag‐tagged) were transfected into 293T cells via a plasmid. The plasmid was synthesized by Mailgene Biosciences Co., Ltd., China. Human WT or K263R mutant Ezrin (3‐flag‐tagged) was transfected into HK‐2 cells via a lentivirus synthesized by Mailgene Biosciences Co., Ltd., China. The cell clones were screened with 1 µg mL^−1^ of purinomycin.

### Animal Experiments

The Peking University Third Hospital Committee on Animal Care approved all experiments on mice. The 8‐week‐old male C57BL/6J mice were purchased from Beijing Vital River Laboratory Animal Technology Co., Ltd. (China) and were bred and housed according to the Guide for the Care and Use of Laboratory Animals published by the National Institutes of Health (NIH Publication, 8th Edition, 2011). The CLP procedure was performed as described previously.^[^ ^73^ ^]^ After isoflurane anesthesia, the cecum was exposed via a 1‐cm incision in the midline of the abdomen. The cecum was ligated with 4‐0 silk between the 3rd and 4th vessel arcades and punctured twice with a 25‐gauge needle. A small amount of feces was extruded (approximately 1 mm in length). Sham operation mice served as controls. After surgery, the incision was sutured in two layers and resuscitated by subcutaneous injection of 1 mL of 0.9% sterile saline solution. Placing the mice on a heated mat helped with recovery. Kidneys were collected at 24 h from CLP mice for the further experiments. The experiment was divided into four groups (n = 24): Sham group, CLP group, CLP+BAY‐876 group which were administered BAY‐876 (5 mg k^−1^g body weight; first gavage 24 h before surgery) at 6 h after CLP surgery, and CLP+LAC group used lactate (pH 6.8, 0.5 g kg^−1^ body weight) at 6 h after CLP surgery. Serum was collected from kidney tissues and peripheral blood for further experiments.

### Proximal Convoluted Tubule Epithelial Cells were Isolated from Adult Mouse Kidneys

The kidney was extracted aseptically after cardiac perfusion with cold saline and immediately transferred to a pre‐cooled PBS buffer. The renal envelope was removed, and the renal cortex was separated and cut to obtain sections with dimensions of approximately 1 mm^3^. The sections were transferred to Hanks solution containing 1 g L^−1^ type I collagenase (Lablead, China, V0891‐100 mg) and shock digested at 37 °C for 20 min. The digestion process was repeated. The digested cell suspension was filtered through 100 mesh and 150 mesh screens, centrifuged at 900 r min^−1^ for 5 min, and washed with PBS thrice. The precipitate was suspended with the pre‐prepared 45% Percoll separation solution (Cytiva, 17 089 102) and centrifuged at 13 000 r min^−1^ at 4 °C for 30 min in an ultra‐fast refrigerated centrifuge. After centrifugation, it was roughly divided into F1‐F4 layers from top to bottom. The F4 layer was isolated, and the proximal renal tubular epithelial cell segment was purified. The segment was washed thrice with PBS, and centrifuged at 900 r min^−1^ for 5 min. The precipitate was collected, and the CUT&Tag test was conducted immediately.

After isolating proximal tubule epithelial cells from mouse kidneys, to assess cell purity, the MRPTEpiC were characterized based on Cytokeratin 18 (CK18) expression. Cell climbing slices were prepared from the extracted MRPTEpiC and subjected to immunofluorescence staining. The primary antibody used was Rabbit Anti‐CK18 Monoclonal Antibody (1:100), with PBS serving as the negative control. In addition, according to literature reports, renal tubular epithelial cells exhibit strong alkaline phosphatase (ALP) activity along the brush border.^[^ ^74^ ^]^ We employed the ALP staining kit (Nanjing Jiancheng Bioengineering Institute, China, D001‐2) to perform ALP evaluation in cell climbing slices.

### Cytometric Bead Array

Serum cytokine concentrations were measured using a Mouse Inflammation Panel (13‐plex) with a V‐bottom Plate (LEGENDplex, 740 446) according to the manufacturer's instructions. Data were analyzed using LEGENDplex data analysis software.

### Histopathology, HE Staining, and Immunohistochemistry of Paraffin Samples

Mouse kidney tissue was fixed overnight in 4% formalin. The sample was embedded in paraffin wax and sliced to obtain 5‐µm‐thick sections. Paraffin sections were washed after dewaxing. Hematoxylin and eosin (HE) stained with hematoxylin‐eosin, dehydrated, and sealed. Immunohistochemical tests were performed with 30% goat serum blocking at 4 °C for 25 min, and the antigen was repaired by hot intercalation with citrate buffer (pH 6.0). Anti‐Pan Kla, H3K18la, and RhoA were used for overnight staining at 4 degrees. The goat anti‐rabbit biotin secondary antibody was used to detect the primary antibody, and the DAB substrate reagent was added for direct color development. After sealing, Pannoramic MIDI (3D HISTECH) was used to collect images.

### Statistical Analysis

Statistical analysis of all experiments was performed using GraphPad Prism 9 software (La Jolla, CA, USA). Data were presented as the mean±standard error from three independent experiments. Student's *t*‐test and One‐way ANOVA followed by Tukey's test were used for comparison. Statistical parameters can be found in numbers and graphical legends. Significant were set as *p* < 0.05.

## Conflict of Interest

The authors declare no conflict of interest.

## Author Contributions

Conceptualization performed by J.Q., B.‐X.Y, and Q.Z.; data curation performed by J.Q. and Y.T.; formal analysis performed by J.Q. and H.‐C.L.; investigation performed by J.Q., Y.T, Q.L., W.‐Y.S., and Z.‐X.L.; methodology performed by J.Q., Q.‐C.W., and W.‐M.F.; project administration performed by S.Y. and L.‐Y.C.; resources validated by S.Y. and L.‐Y.C.; software validated by J.Q. and H.‐C.L.; supervision performed by S.Y. and L.‐Y.C.; validation performed by J.Q., Q.‐C.W., and W.‐M.F.; visualization acquired by J.Q., S.Y., and L.‐Y.C.; writing – original draft done by J.Q; Writing – review & editing performed by S.Y. and L.‐Y.C.

## Supporting information

Supporting Information

## Data Availability

The data that support the findings of this study are available from the corresponding author upon reasonable request.
