# Supplementary material for: Histone H3K18 and Ezrin Lactylation Promote Renal Dysfunction in Sepsis‐Associated Acute Kidney Injury
Source: Adv Sci (Weinh). 2024 May 20;11(28):2307216. doi: 10.1002/advs.202307216 (PMC11267308; doi:10.1002/advs.202307216)
Supplement: Supplementary file 1 — Supporting Information [file ADVS-11-2307216-s001.docx]

Supplementary Materials for

**Histone H3K18 and Ezrin Lactylation Promote Renal Dysfunction in**

**Sepsis-associated****Acute Kidney Injury**

Jiao Qiao *et al.*

**This PDF file includes:**

Figs. S1 to S5

Tables S1

**Supplementary Figures**

**
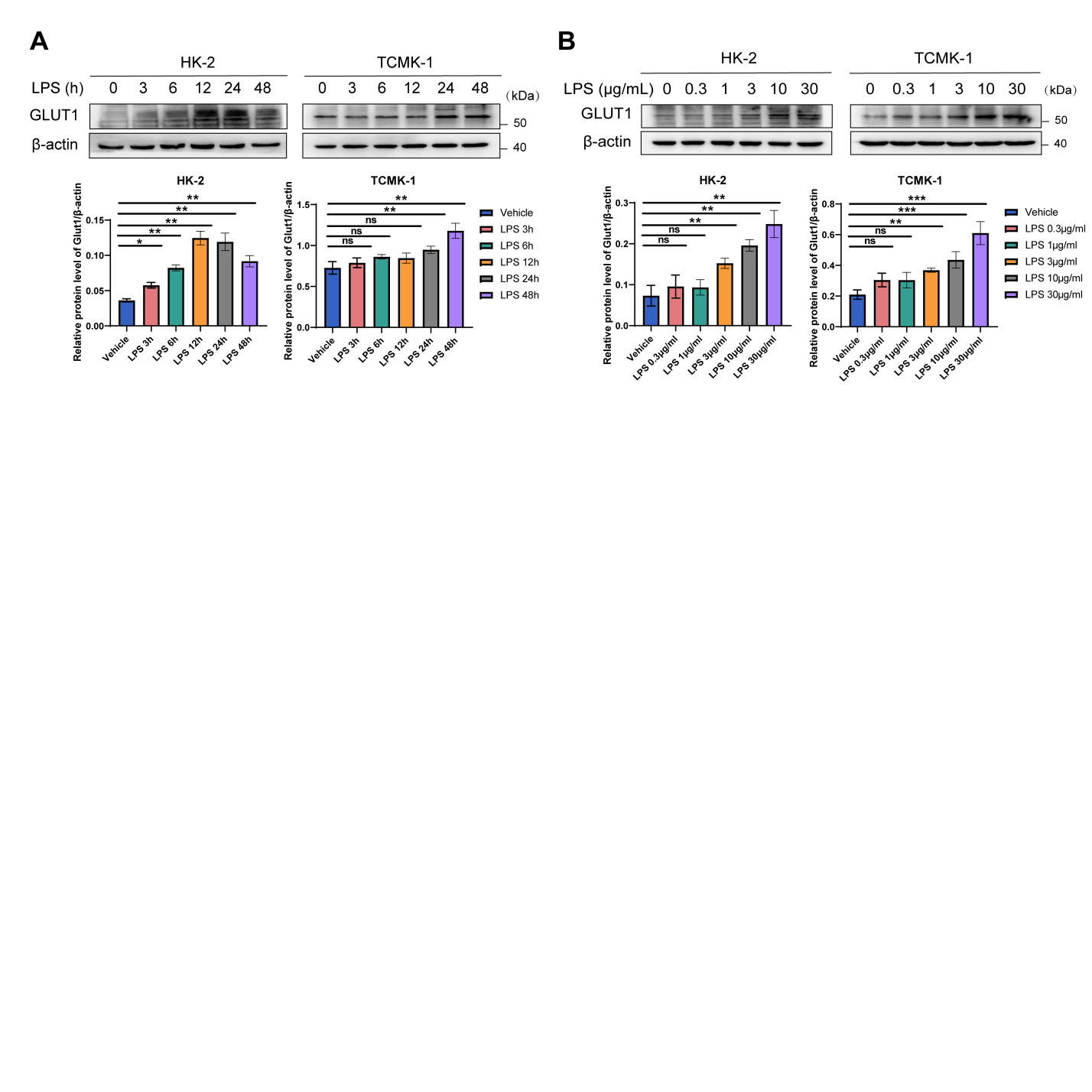
**

**Figure S1. LPS induced increased GLUT1 expression in renal tubular epithelial cells.** (A) Western blotting analysis of GLUT1 protein levels of HK-2 cells and TCMI-1 cells at 0, 3, 6, 12, 24, and 48-hour after 10 μg/mL LPS treatment. (B) Western blotting analysis of GLUT1 protein levels of HK-2 cells and TCMI-1 cells treated with 24-hour LPS of 0, 0.3, 1, 3, 10, and 30 μg/mL.**P* < 0.05, ** *P* < 0.01, and *** *P* < 0.01; ns, not significant.


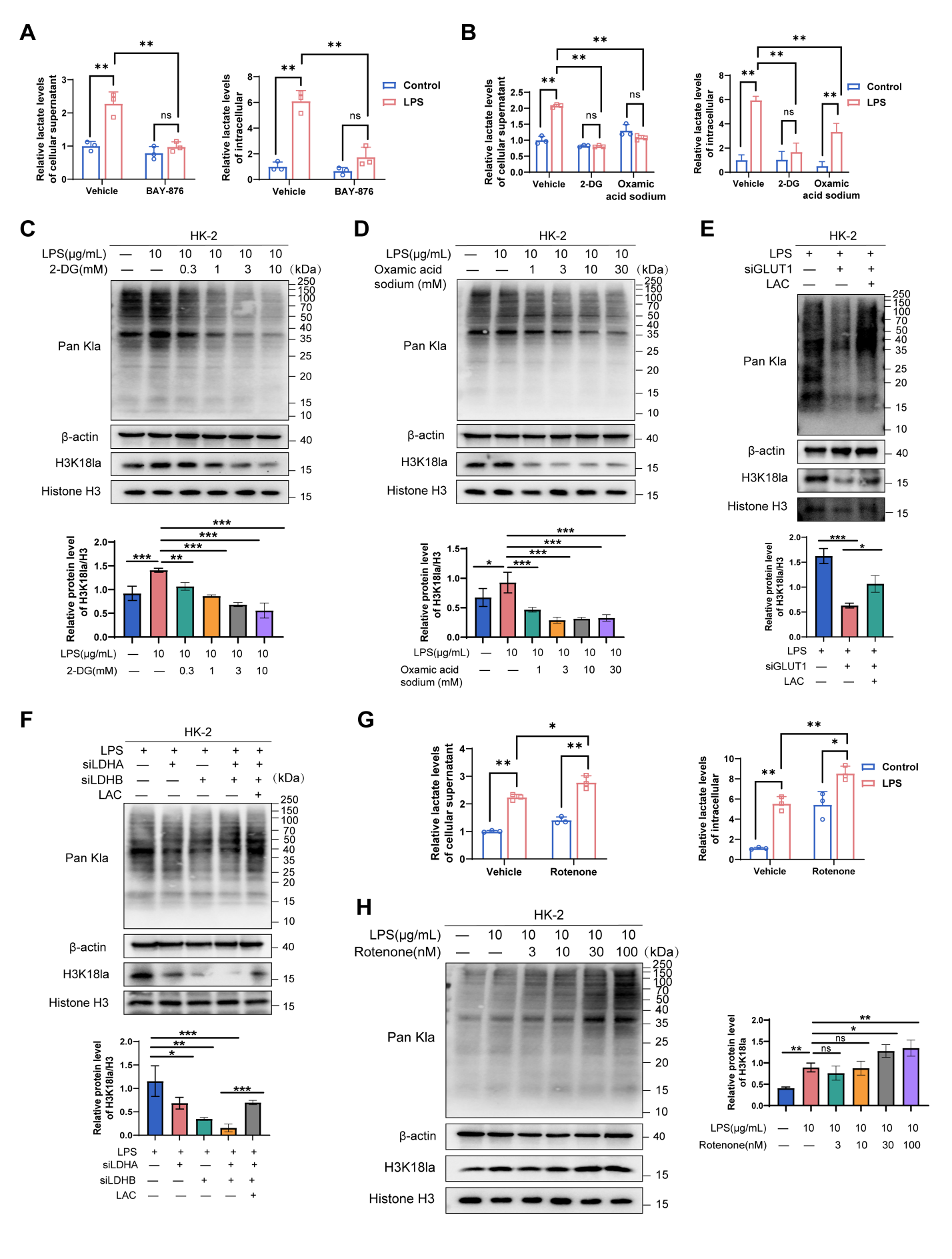


**Figure S2. Inhibition of glycolysis can decrease the level of histone lactylation and inhibit LPS-induced RTECs injury.** (A, B) The relative levels of lactate in the supernatant and intracellular of HK-2 cells cultured in 10 μg/mL LPS±1μM BAY-876 (A) or 2 mM 2-DG or 10mM Oxamic acid sodium (B) for 24 h , n = 3 independent experiments. (C, D) Western blotting analysis of Pan Kla and H3K18la in HK-2 cells after treated with different concentrations of 2-DG (C) or Oxamic acid sodium (D) and 10 μg/mL LPS. (E, F) Western blotting analysis of Pan Kla and H3K18la in HK-2 cells after knockdown with GLUT1 (E) or LDHA/LDHB (F) with or without lactate. (G) The relative levels of lactate in the supernatant and intracellular of HK-2 cells treated with 10 μg/mL LPS±30mM Rotenone for 24 h, n = 3 independent experiments. (H) Western blotting analysis of Pan Kla and H3K18la in HK-2 cells after treated with different concentrations of Rotenone and 10 μg/mL LPS. n = 3 independent experiments. **P* < 0.05, ** *P* < 0.01, *** *P* < 0.01; ns, not significant vs. LPS group.


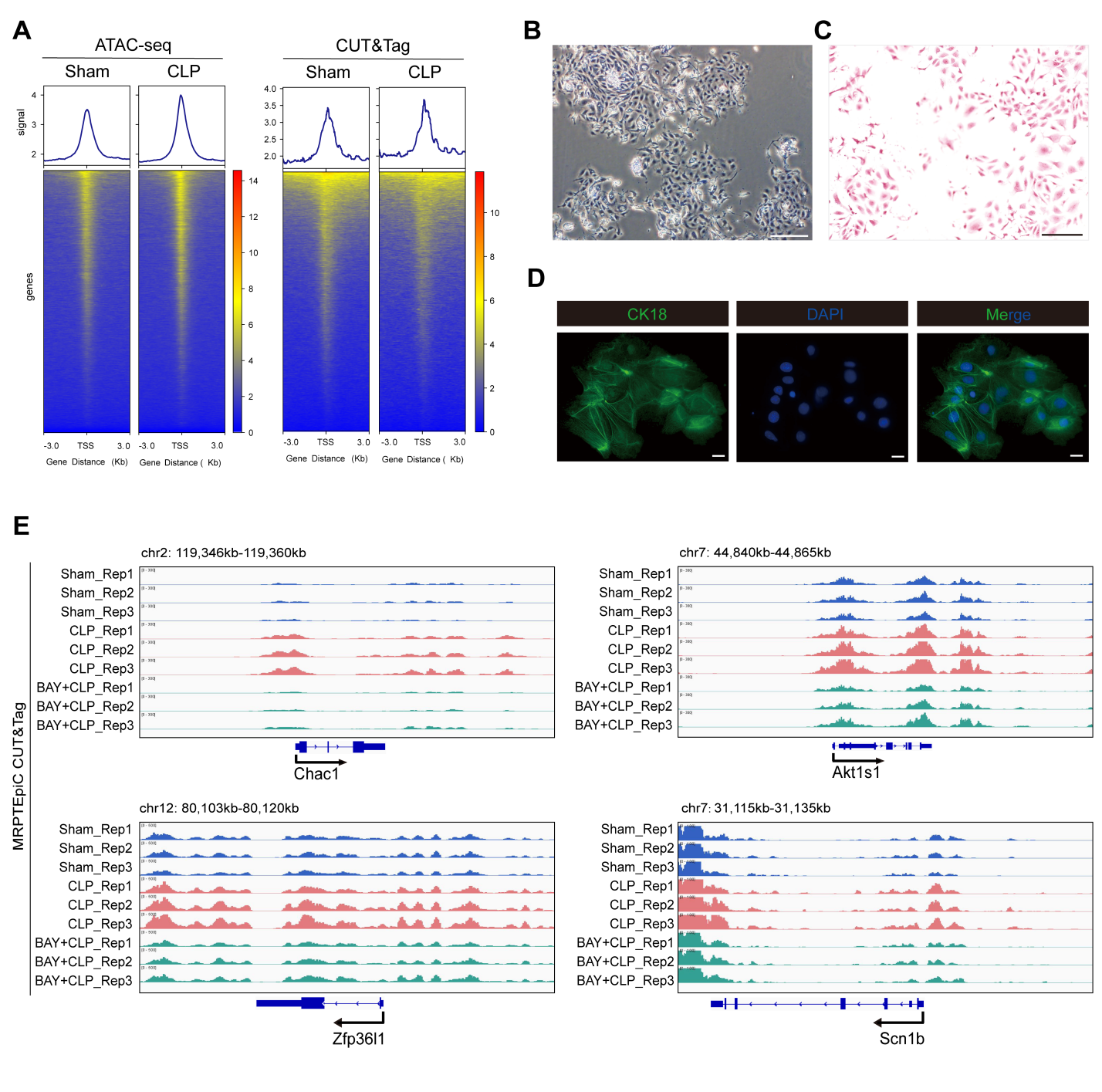


**Figure S3. Identification of MRPTEpiC cells and bioinformatics analysis.** (A) Heatmaps of ATAC (left) and H318la CUT&Tag (right) signals were visualized by deepTools in Sham and CLP group. The findings are ordered by signal strength. (B) Image of MRPTEpiC cell culture status after 72 hours of isolation and cultivation (scale bar=100 µm). (C) Alkaline phosphatase (ALP) activity test (scale bar=100 µm). (D) Immunofluorescence experiment on cell climbing slices to detect the expression of CK18 in primary cultured MRPTEpiC (scale bar=20 µm). CK18 was stained with phalloidin (green), and nuclei were stained with DAPI (blue). (E) IGV tracks for Chac1, Akt1s1, Zfp36l1, and Scn1b from CUT&Tag analysis.


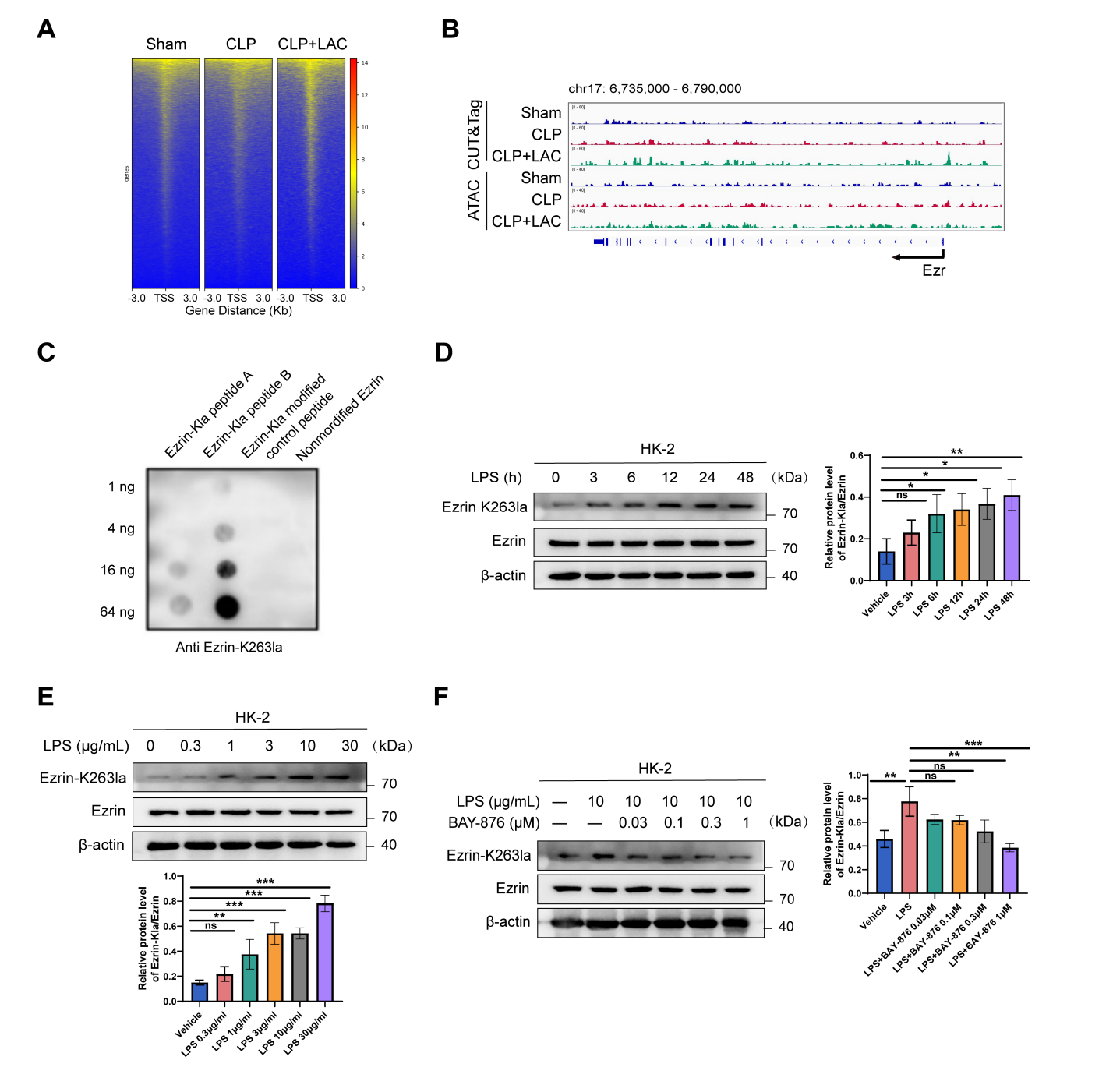


**Figure S4. Identification of MRPTEpiC cells and CUT&Tag analysis.** (A) Heatmaps for H3K18la binding peaks in mice from the Sham, CLP, and CLP+LAC groups. The color depth indicates the relative number of reads, and genes were clustered together to show the binding trends of lactylation modifications corresponding to all genes. (B) IGV tracks for Ezr from ATAC-seq and CUT&Tag analysis. (C) Dot blot assay for detection of Anti-Lactyl-Ezrin (K263) antibody. (D) Western blotting analysis of Ezrin-K263la level of HK-2 cells at 0, 3, 6, 12, 24, and 48-hour after 10 μg/mL LPS treatment. (E) Western blotting analysis of Ezrin-K263la level of HK-2 cells treated with 24-hour LPS of 0, 0.3, 1, 3, 10, and 30 μg/mL. (F) Western blotting analysis of Ezrin-K263la level in HK-2 cells after treated with different concentrations of BAY-876 and 10 μg/mL LPS.


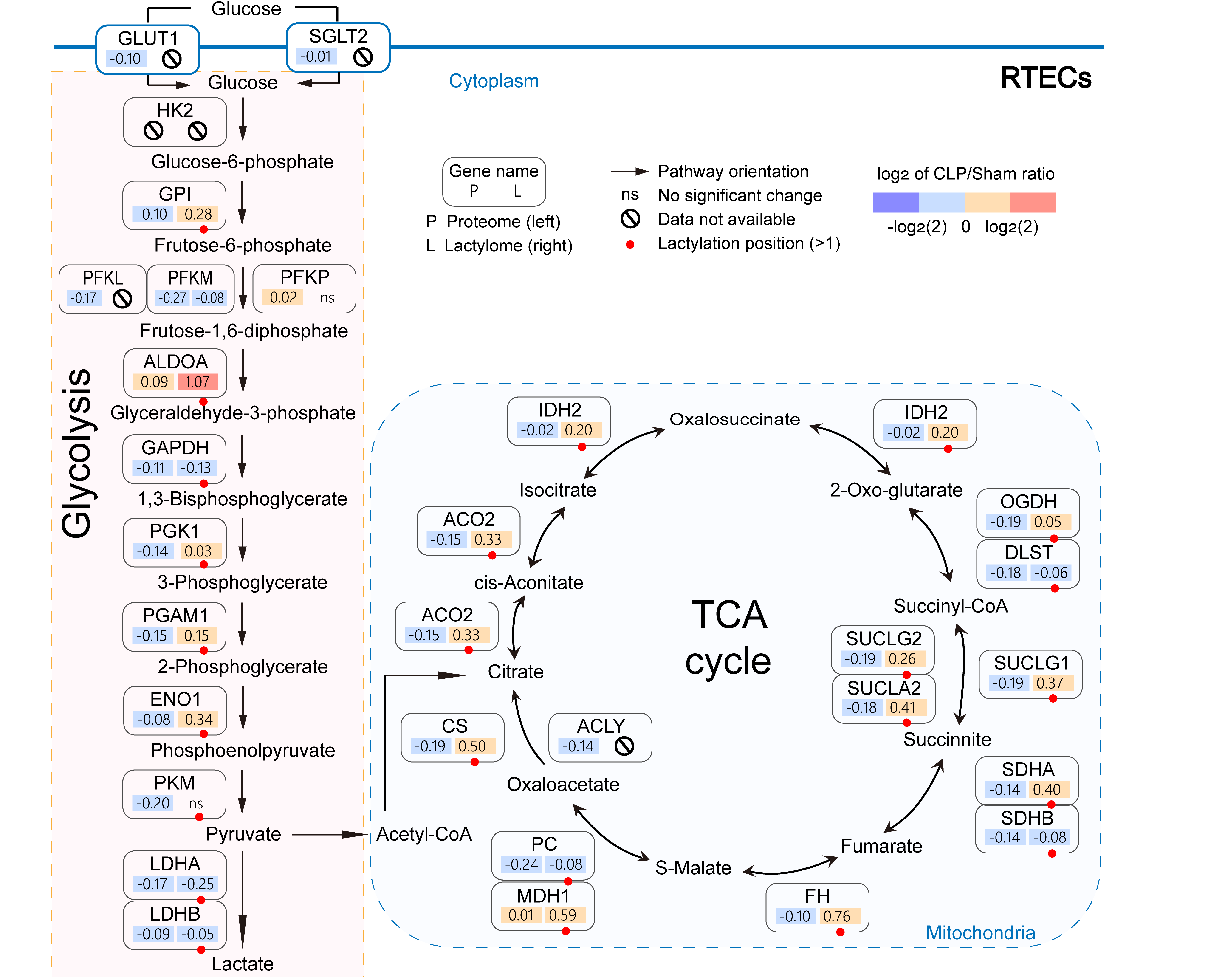


**Figure S5. Proteomics and lactate modification of key enzymes of glycolysis and tricarboxylic acid cycle in SA-AKI renal tissue.**

**Supplemental Table**

**Tables S1. Primers used for qRT-PCR analysis.**

| Gene | Sequences (5’-3’) |
| --- | --- |
| IL-1β-Forward | CACCTCTCAAGCAGAGCACAG |
| IL-1β-Reverse | GGGTTCCATGGTGAAGTCAAC |
| IL-6-Forward | TGAGGAGACTTGCCTGGTGA |
| IL-6-Reverse | TGAGGAGACTTGCCTGGTGA |
| TNF-α-Forward | CCAGGAGAAAGTCAGCCTCCT |
| TNF-α-Reverse | CCAGGAGAAAGTCAGCCTCCT |
| β-actin-Forward | CATGTACGTTGCTATCCAGGC |
| β-actin-Reverse | CATGTACGTTGCTATCCAGGC |
